# The Endo-α(1,4) Specific Fucoidanase Fhf2 From *Formosa haliotis* Releases Highly Sulfated Fucoidan Oligosaccharides

**DOI:** 10.3389/fpls.2022.823668

**Published:** 2022-02-02

**Authors:** Vo Thi Dieu Trang, Maria Dalgaard Mikkelsen, Marlene Vuillemin, Sebastian Meier, Hang Thi Thuy Cao, Jan Muschiol, Valentina Perna, Thuan Thi Nguyen, Vy Ha Nguyen Tran, Jesper Holck, Tran Thi Thanh Van, Huynh Hoang Nhu Khanh, Anne S. Meyer

**Affiliations:** ^1^Protein Chemistry and Enzyme Technology Section, Department of Biotechnology and Biomedicine, Technical University of Denmark, Kongens Lyngby, Denmark; ^2^NhaTrang Institute of Technology Research and Application, Vietnam Academy of Science and Technology, Nha Trang, Vietnam; ^3^Department of Chemistry, Technical University of Denmark, Kongens Lyngby, Denmark; ^4^Ocean EcoSystems Biology Unit, GEOMAR Helmholtz Centre for Ocean Research Kiel, Kiel, Germany

**Keywords:** FTIR, *Fucus evanescens*, *Sargassum mcclurei*, calcium dependency, T9SS, sulfation

## Abstract

Fucoidanases are endo-fucoidanases (also known as endo-fucanases) that catalyze hydrolysis of α-glycosidic linkages in fucoidans, a family of sulfated fucose-rich polysaccharides primarily found in the cell walls of brown seaweeds. Fucoidanases are promising tools for producing bioactive fucoidan oligosaccharides for a range of biomedical applications. High sulfation degree has been linked to high bioactivity of fucoidans. In this study, a novel fucoidanase, Fhf2, was identified in the genome of the aerobic, Gram-negative marine bacterium *Formosa haliotis*. Fhf2 was found to share sequence similarity to known endo-α(1,4)-fucoidanases (EC 3.2.1.212) from glycoside hydrolase family 107. A C-terminal deletion mutant Fhf2∆484, devoid of 484 amino acids at the C-terminus, with a molecular weight of approximately 46 kDa, was constructed and found to be more stable than the full-length Fhf2 protein. Fhf2∆484 showed endo-fucoidanase activity on fucoidans from different seaweed species including *Fucus evanescens*, *Fucus vesiculosus*, *Sargassum mcclurei*, and *Sargassum polycystum*. The highest activity was observed on fucoidan from *F. evanescens*. The Fhf2∆484 enzyme was active at 20–45°C and at pH 6–9 and had optimal activity at 37°C and pH 8. Additionally, Fhf2∆484 was found to be calcium-dependent. NMR analysis showed that Fhf2∆484 catalyzed hydrolysis of α(1,4) linkages between L-fucosyl moieties sulfated on C2 (similar to Fhf1 from *Formosa haliotis*), but Fhf2∆484 in addition released oligosaccharides containing a substantial amount of 2,4-disulfated fucose residues. The data thus suggest that the Fhf2∆484 enzyme could be a valuable candidate for producing highly sulfated oligosaccharides applicable for fucoidan bioactivity investigations.

## Introduction

Fucoidans are fucose-rich sulfated polysaccharides found primarily in the cell walls of brown seaweeds. Depending on the brown algae species, fucoidans present a high structural diversity and vary in monomer composition, type of linkages, sulfation pattern, and degree of branching and of acetylation (Ale and Meyer, 2013). Fucoidans have received increased attention over the past years due to their wide range of biological activities, such as anticancer, anticoagulant, anti-tumor, antioxidant, anti-inflammatory, anti-thrombotic, and immunomodulatory effects (Wang et al., 2019) that provide a promising potential for application of fucoidans in food, cosmetics, nutrition, and pharma. The bioactivities of fucoidans are tightly linked to their structure, including degree of sulfation, as well as the molecular weight (Ale et al., 2011a,b).

Based on their backbone structures, fucoidans can be categorized as α(1,3)-L-fucans, α(1,3)/(1,4)-L-fucans, or sulfated galactofucans (Li et al., 2008). The fucoidans from the α(1,3)-L-fucans group consist of a backbone of α-L-fucosyl (L-fucopyranosyl) residues linked through α(1,3)-O-glycosidic bonds. This group includes fucoidans from *Saccharina cichorioides* (Zvyagintseva et al., 2003; Anastyuk et al., 2010), *Saccharina latissima* (previously *Laminaria saccharina*; Bilan et al., 2010), and *Lessonia vadose* (Chandía and Matsuhiro, 2008). The α(1,3)/(1,4)-L-fucans have a backbone of alternating α(1,3) and α(1,4) linked fucosyl residues (Patankar et al., 1993; Bilan et al., 2002). These fucoidans are found in species, such as *Fucus vesiculosus* and *Fucus evanescens,* and the fucopyranosyl residues in fucoidans from these seaweed species can be sulfated at C2/C3/C4 and can moreover be acetylated and have short branches (Patankar et al., 1993; Bilan et al., 2002).

Fucoidan structures from *F. vesiculosus* and *F. evanescens* differ by the degree and the position of sulfate substitutions, which are mainly found at C2 and at C2/C4 (Patankar et al., 1993; Bilan et al., 2002; Menshova et al., 2016). C3 sulfation is rare in the fucoidan in *F. evanescens* (Menshova et al., 2016), while the fucoidan from *F. vesiculosus* is more highly sulfated and may contain sulfate substitutions at C2, C2/C3, C2/C4 (Patankar et al., 1993). The most structurally diverse group of fucoidans are the sulfated galactofucans that present complex structures with a high content of fucose and galactose residues with varying ratios and types of glycosidic linkages. Sulfated galactofucans are mainly found in *Sargassum* and *Turbinaria* species, such as *Sargassum mcclurei*, *Sargassum polycystum,* and *Turbinaria ornata*, where the sulfate groups are positioned at C2 and/or C4 of the backbone fucosyl residues (Bilan et al., 2013; Thinh et al., 2013; Ermakova et al., 2016).

Highly sulfated fucoidan from *F. evanescens* was demonstrated to have higher anti-angiogenic and anti-osteogenic activity (Ohmes et al., 2020). In addition, high sulfation degree of fucoidan was found to enhance anti-angiogenic and anti-tumor activities (Koyanagi et al., 2003). Low molecular weight fucoidans are more soluble, exhibit higher molecular mobility and diffuse more easily into the cell membrane (Yang et al., 2008; Torres and Díaz, 2020), and have generally been associated with improved anticancer activities compared to high molecular weight fucoidans (Yang et al., 2008; Cho et al., 2011; Chen et al., 2015). Due to their catalytic selectivity, enzymes are attractive as tools for gentle extraction of fucoidan, retaining the sulfations (Nguyen et al., 2020) and for selective modification of fucoidans to tailor-make sulfated, low molecular weight fucoidans having potentially high bioactivity (Silchenko et al., 2013, 2018; Cao et al., 2018; Vuillemin et al., 2020; Zueva et al., 2020).

Based on the similarities of amino acid sequences, secondary structures, or glycosidic bond specificity, most known fucoidanases have been classified into the glycosyl hydrolase (GH) families GH107 and GH168 in the CAZy database (Lombard et al., 2014). Endo-fucoidanases (endo-fucanases) that catalyze cleavage of α(1,4) glycosidic bonds in fucoidans are classified in GH family 107 and as EC 3.2.1.212, while family GH168 endo-fucoidanasas are known as endo-α(1,3)-L fucanases (EC 3.2.1.211). Currently, 47 putative fucoidanases are classified in family GH168, and only one of these enzymes has been characterized, while 28 putative fucoidanases have been identified in GH107 with only six of them being characterized. Five GH107 fucoidanases, FWf1 and FWf2 from *Wenyingzhuangia fucanilytica* CZ1127 (Zueva et al., 2020), FFA1 and FFA2 from *Formosa algae* KM3553 (Silchenko et al., 2013, 2017a,b), and Fhf1 from the marine bacterium *Formosa haliotis* (Vuillemin et al., 2020) have all been characterized, but not yet been included in the CAZy database.

The extended C-terminus of several GH107 enzymes does not seem to be important for the catalytic function of the enzymes and can readily be deleted to produce truncated, stabilized, active enzymes that are not destined for degradation during heterologous expression in *Escherichia coli*. This is notably the case for the recombinant fucoidanases MfFcnA, Fda1, and Fda2 from *Alteromonas* sp. SN-1009 and most recently Fhf1 (Colin et al., 2006; Cao et al., 2018; Vuillemin et al., 2020).

In the present study, we report the characterization of a novel GH107 fucoidanase, Fhf2, identified in the genome of *F. haliotis* that was isolated from the gut of the abalone *Haliotis gigantea* (Tanaka et al., 2015). The expression, purification, stabilization, and substrate specificity of the recombinant enzyme, Fhf2Δ484, obtained by targeted C-terminal truncation is described. Fhf2Δ484 is shown to be specific for α(1,4)-linkages between C2 sulfated fucosyl residues in fucoidans from *F. evanescens* and to release fucoidan oligosaccharides with C2 sulfation and in addition allow for C2,4-disulfated fucose residues positioned internally likely in longer oligosaccharides, unlike other characterized endo-fucoidanase enzymes to date.

## Materials and Methods

### Seaweeds and Fucoidan Substrates

Crude fucoidans were extracted, as previously described, from *S. mcclurei* (Thinh et al., 2013), *S. polycystum* (Bilan et al., 2013), and *T. ornata* (Thanh et al., 2013). They were further fractionated by ion-exchange chromatography (Bilan et al., 2013). The fucoidans from *F. evanescens* and *S. latissima* were extracted, purified, and fractionated, using an enzyme-assisted extraction method as previously described (Nguyen et al., 2020; fraction 3 from *S. latissima* and fraction 2 from *F. evanescens* was used). Fucoidan from *F. vesiculosus* (F8190) was purchased from Sigma-Aldrich (Steinheim, Germany), and used as is.

### Identification of the *fhf2* Gene, Sequence Analysis, and 3D Structure Modeling

The gene encoding the fucoidanase Fhf2 (NCBI accession: WP_066217784.1) was identified in the genome of *F. haliotis* (Genbank: BDEL00000000) by BLAST using known fucoidanase-encoding genes (encoding family GH107 fucoidanases). A signal peptide was predicted by using the SignalP 5.0 server.^1^ Domains were predicted in the Fhf2 protein using InterProScan.^2^

Protein sequence comparison was performed using protein Blast (NCBI). For comparisons, the following sequences were used: D1 sequence of fucoidanase Fhf1 (WP066217780.1); FFA1 (WP057784217.1) and FFA2 (WP057784219.1); MfFcnA (CAI47003.1); FcnA_5A (AYF59291.1) from *Psychromonas* sp. SW5A; FcnA_19D (AYF59292.1) from *Psychromonas* sp. SW19D; AXE80_07420 (ANW96115.1), AXE80_07425 (ANW96116.1), AXE80_07310 (ANW96098.1) and AXE80_07305 (ANW96097.1) from *Wenyingzhuangia fucanilytica* CZ1127; D1818_06650 (AXT50524.1) and D1818_06655 (AXT50525.1) from *Aquimarina* sp. BL5; Fp273 (AYC81238.1), Fp277 (AYC81239.1) and Fp279 (AYC81240.1) from uncultured bacteria from an environmental sample; FNB79_00785 (QDO92576.1) from *Formosa sediminum* PS13; Fleli_2704 (AFM05060.1) from *Bernardetia litoralis* DSM6794; SVI_0379 (BAJ00350.1) from *Shewanella violacea* DSS12; Fda1 (AAO00508.1), Fda2 (AAO00509.1) from *Alteromonas* sp. SN-1009. CLC Genomics workbench program version 8.0 (Qiagen, https://digitalinsights.qiagen.com) was used as an aid to identify the conserved residues in the protein sequences.

A 3D homology model of Fhf2 was prepared by using YASARA Structure 17.8.15 (YASARA Biosciences GmbH, Vienna, Austria) *via* the built-in homology modeling function. Nine different template structures were identified and manually selected: 6DLH (the MfFcnA fucoidanase), and eight GH29 fucosidase structures 2WSP, 2ZXD, 3GZA, 3UET, 4OUE, 4PSR, 4ZRX, and 5K9H. The program automatically identified two additional template structures: 6DMS (FcnA_H294Q mutant) and 5HFS (Gingipain R2 from *Porphyromonas gingivalis*). After preparing 52 models using all templates, YASARA prepared a hybrid model, which was selected for refinement using the md_refine macro as supplied with the YASARA Structure package. The final models were visually presented using PyMOL (The PyMOL Molecular Graphics System, version 2.2.0 Schrodinger L.L.C., Cambridge, MA, United States). The electrostatic surface of the Fhf2 homology model was calculated using the Particle Mesh Ewald (ESPPME) approach (Essmann et al., 1995) in YASARA 20.4.24 (Krieger and Vriend, 2014). For coloring of the surface, a maximum electrostatic potential (ESP) of 100 kJ/mol was used.

### Gene Constructs and Cloning

The *fhf2* gene from *F. haliotis*, lacking the predicted N-terminal predicted signal peptide and containing a C-terminal 6xHis-tag was synthesized codon-optimized for *E. coli* expression and subcloned into the pET31b(+) vector between the NdeI and XhoI restriction sites (Thermo Fisher Scientific, Waltham, MA, United States). The C-terminally truncated version *fhf2Δ484* was obtained by removing the last 484 amino acids, corresponding to the two predicted CBMs, and then, a C-terminal 10xHis-tag was added. The *fhf2Δ484* gene was amplified by polymerase chain reaction (PCR) using CloneAmp HiFi polymerase premix (Takara Bio USA, Inc., Mountain View, CA, United States). Using 5ʹ-CATATGCAACAAATACCCGATCCAG-3′ as forward primer and 5ʹ-CAGTCATCTCGAGCTAATGGTGATGGTGATGGTGCGGCGCACCCGGATATTGGTTAAC-3′ as reverse primer. The PCR products were digested with the restriction enzyme DpnI at 37°C overnight and then purified by GFX™ PCR DNA Purification Kit (G.E. Healthcare, Uppsala, Sweden). The purified PCR product and pET31b (+) vector was digested with NdeI and XhoI restriction enzymes for 4 h at 37°C and ligated together with an insert:vector of mass ratio of 3:1 using T4 DNA ligase (Thermo Fisher Scientific, Waltham, MA, United States) at 16°C, overnight. The ligation product was used to transform *E. coli* DH5α as plasmid propagation host (Invitrogen® Life Technologies, Thermo Fisher Scientific, Waltham, MA, United States). Positive transformants were selected on LB ampicillin plates. Plasmids pET31b(+)_*fhf2Δ484* were then extracted and checked by sequencing (Macrogen Europe, Amsterdam, Netherlands).

### Production and Purification of Recombinant Fucoidanase

Fhf2 and Fhf2∆484 were expressed in *E. coli* BL21 (DE3) harboring the Pch2 (pGro7) plasmid (Takara Biolabs, Göteborg, Sweden) and purified on Ni^2+^ Sepharose resin (G.E. Healthcare, Chicago, IL, United States) as previously described for Fhf1 (Vuillemin et al., 2020). The purified proteins were desalted on PD-10 desalting columns (G.E. Healthcare, Uppsala, Sweden) equilibrated with buffer (20 mM Tris-HCl buffer, 250 mM NaCl, pH 7.4) at 4°C. The molecular weight and purity of the protein in the eluted fractions were estimated by the sodium dodecyl sulfate-polyacrylamide gel electrophoresis assay (SDS-PAGE) and Western blotting assay using poly-his antibodies (Sigma-Aldrich, Steinheim, Germany). Protein content was determined by the Bradford method using the protein assay reagent (Bio-Rad, CA, United States) with bovine serum albumin as the standard (Bradford, 1976). The protein markers were Precision Plus Protein standard for SDS-PAGE, Precision Plus Protein Dual Color Standard for Western Blots (Bio-Rad, Hercules, CA, United States).

### Enzymatic Assays for C-PAGE

The fucoidanase activity was assayed using 0.9% (w/v) fucoidan substrate, 0.3 mg/ml enzyme, 10 mM Tris-HCl buffer pH 7.4–8, 100–125 mM NaCl and 10 mM CaCl_2_ and was incubated for 4 h at 35–37°C. The reaction was stopped by incubating at 80°C for 5 min and further analyzed by C-PAGE. For assessment of optimal enzyme conditions, the varying conditions were changed accordingly.

For determining the specific substrate specificity, experiments were performed with different fucoidans from *F. evanescens*, *F. vesiculosus*, *S. mcclurei*, *S. polycystum*, *T. ornata*, and *S. latissima* at pH 7.4 and 24h incubation time.

The effects of divalent cations were investigated by removing any bound divalent cations from the Fhf2Δ484 by incubating the enzyme with 2 mM EDTA at room temperature for 4 h. EDTA was removed by desalting on a PD10 column. The assay reaction was performed with 10 mM of the different divalent cations Ca^2+^, Mg^2+^, Mn^2+^, Cu^2+^, Fe^2+^, Zn^2+^, Co^2+^, and Ni^2+^, respectively. The thermal stability was evaluated by incubating Fhf2Δ484 at 37, 40, and 45°C without substrate and then performing C-PAGE at set sampling times to assess the residual activity.

### Carbohydrate Polyacrylamide Gel Electrophoresis

10 μl sample were mixed with 10 μl loading buffer (20% glycerol and 0.02% phenol red in water). 6 μl of sample were electrophoresed through a 20% resolving polyacrylamide gel with 100 mM Tris-borate buffer pH 8.3 for 2 h at 25 mA. Gel staining was performed in two steps: First with a solution containing 0.05% alcian blue 8GX (Panreac, Barcelona, Spain) in 2% acetic acid for 60 min and then with 0.01% O-toluidine blue (Sigma-Aldrich, Steinheim, Germany) in 50% aqueous ethanol and 1% acid acetic for 30 min. The gel was destained by washing with water. The oligosaccharide standard (St) was obtained after enzymatic reaction of 1% (w/v) *F. evanescens* fucoidan (not de-acetylated) using 0.3 mg/ml FFA2 from *F. algae*. The lowest band of a similarly treated standard using de-acetylated *F. evanescens* fucoidan corresponds to a tetra-saccharide of (1,4) and (1,3)-linked α-L-fucosyls with each fucosyl residue sulfated at C2 (Silchenko et al., 2017b).

### Assessment of Molecular Weight Distribution of Fucoidan Structures

The molecular weight distribution of the native and hydrolyzed fucoidan was estimated by high-performance size exclusion chromatography (HPSEC) using an Ultimate iso-3100 SD pump with a WPS-3000 sampler (Thermo Scientific, Waltham, MA, United States) connected to an ERC RefractoMax 520 refractive index detector (Thermo Scientific, Waltham, MA, United States). 100 μl of sample was loaded on a Shodex SB-806 HQ column (300 × 8 mm) equipped with a Shodex SB-G guard column (50 mm × 6 mm; Showa Denko K.K., Tokyo, Japan).

Elution was performed with 100 mM sodium acetate pH 6 at a flow rate of 0.5 ml/min at 40°C. External pullulan standards (PSS Polymer Standards Service GmbH, Mainz, Germany) were applied to establish a polynomial relationship between the logarithmic molecular weight and the corresponding retention times separating the polymer molecules in order to convert the retention times of the samples to molecular weights. Data visualization was performed in Python 3.8.

### Thermal Unfolding and Melting Temperature Determination

5 μM of purified enzyme in 20 mM Tris-HCl, 250 mM NaCl, pH 7.4 was prepared for the thermal unfolding experiment. The sample was loaded into a UV capillary (Prometheus NT.48). The experiments were performed with an increasing temperature gradient at 1°C per min from 20 to 95°C using a Nanotemper Prometheus NT.48 machine (NanoTemper technologies, Munich, Germany). Protein unfolding was measured as change in tryptophan fluorescence at wavelengths 330 and 350 nm. Melting temperature, T_m_, was determined by detecting the maximum of the first derivative of the fluorescence at 330 and 350 (F330/F350).

### Fourier Transform Infrared Spectroscopy Measurement

A MilkoScan™ FT2 (FOSS ANALYTICAL, Hillerød, Denmark) FTIR instrument was used in the 1,000–2,000 cm^−1^ range to scan all IR spectra with an optical resolution of 14 cm^−1^. The reaction samples (1 ml) included 2% fucoidan *F. evanescens* in 0.02 M Tris-HCl buffer pH 8, 10 mM of CaCl_2_ and different Fhf2Δ484 concentrations at 1.98, 3.30, 4.83, 7.69, 9.45, and 18.02 μM. Each sample was manually injected directly into the cuvette of the FTIR instrument by a single-use syringe.

The cuvette was controlled at 42°C and had a 50 μm path length; 30 consecutive spectra, with recording time per spectrum of 16.6 s, were recorded to obtain spectral evolution profiles (Tran et al., 2021, unpublished). In order to ensure that the measured spectral evolution was due to enzymatic action on the substrate and not due to other any change of elements of the reaction, three different control experiments were performed: (1) the substrate control profile was measured by replacing the enzyme sample with 0.02 M Tris-HCl buffer at pH 8; (2) the enzyme control profile was obtained by replacing the substrate with 0.02 M Tris-HCl buffer at pH 8; and (3) with heat-inactivated enzyme added to the substrate. All reactions and controls were performed in triplicates. The acquired spectral data were exported using Foss Integrator (version 1.5.3, Foss Analytical, Hillerød, Denmark). All subsequent data analysis was carried out by PARAFAC analysis using MATLAB (The MathWorks Inc., MA, United States), the N-Way toolbox (Copenhagen University, Denmark) and the Statistics and Machine learning toolbox (The MathWorks Inc., MA, United States) as previously described (Perna et al., 2019; Tran et al., 2021, unpublished).

### Nuclear Magnetic Resonance Spectroscopy

180 mg of fucoidan from *F. evanescens* (0.9% final concentration) was incubated with 0.3 mg/ml Fhf2Δ484 fucoidanase in 20 mM Tris-HCl buffer, at pH 7 and 37°C and 10 mM CaCl_2_. The enzymatic reaction was stopped after 24 h by incubation at 80°C for 10 min. HMP fucoidan was precipitated by addition of cold ethanol at a ratio of 1:3 (v/v) and incubated at 4°C for 24 h. The high molecular weight reaction products (HMP) and the supernatant containing low molecular weight reaction products (LMP) were separated by centrifugation at 15,000 rpm for 30 min. To ensure that the hydrolysis was completed, additional Fhf2Δ484 fucoidanase was added to the HMP. The reaction products were visualized using C-PAGE. Samples were subsequently lyophilized.

The fucoidan samples (~10 mg) were dissolved in 500 μl ^2^H_2_O, and NMR spectra were collected on an 800 MHz Bruker Avance III instrument equipped with an Oxford magnet and a TCI cryoprobe (5 mm). Specifically, ^1^H 1D NMR spectra (of 16,384 complex data point sampling 1.7 s), ^1^H-^1^H TOCSY (2048 × 256 complex data points sampling 128 and 16 ms in the direct and indirect dimension, respectively), ^1^H-^1^H COSY (2048 × 256 complex data points sampling 128 and 16 ms in the direct and indirect dimension, respectively), ^1^H-^13^C HMBC (2048 × 128 complex data points sampling 256 and 6.3 ms, respectively), and ^1^H-^13^C HSQC (2048 × 512 complex data points sampling 160 and 21.2 ms) were acquired. All NMR spectra were processed with ample zero filling in all dimensions and baseline correction using Bruker Topspin 3.5 pl7 software. The spectra were analyzed using the same software.

## Results

### Sequence Analysis of Fhf2

**Figure 1:**
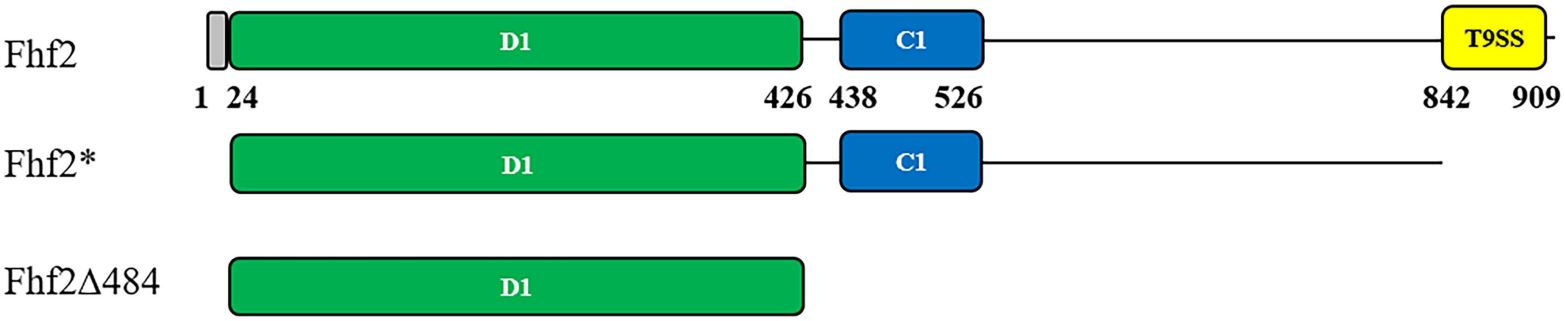
Predicted protein domain structures of Fhf2 and Fhf2Δ484. Numbering corresponds to amino acids in the full-length Fhf2 sequence. Predicted domains are indicated with the amino acid numbers and in colors. Grey: secretion signal peptide, green: D1 domain; cyan: cadherin-like superfamily domain (IPR015919); yellow: a secretion system C-terminal sorting domain (IPR026444). Fhf2* is the full-length gene for heterologous expression in *Escherichia coli* devoid of the N-terminal signal peptide as well as the C-terminal T9SS domain. The C-terminal deletion mutant Fhf2Δ484 only contains the predicted catalytic D1 domain.

Fhf2 (Sequence ID: WP_066217784.1) is a 910 amino acid long protein with a 24 amino acid long predicted N-terminal signal peptide. According to BLASTp analysis, Fhf2 shares highest identity with the previously characterized endo α(1,4)-fucoidanase FFA2 from *Formosa algae* (Sequence ID: WP_057784219.1). Fhf2 shows 82% sequence identity with FFA2 with a 99% query coverage. Two known domains were found in the Fhf2 protein sequence using InterProScan^3^ (Jones et al., 2014; Figure 1). The first domain of 88 amino acids (from amino acid 438 to 526) length was predicted to belong to the cadherin-like domain family (IPR015919) with a Greek key topology Ig-like beta-sandwich structure (Dickens et al., 2002). This domain belongs to a large family of calcium-dependent cell adhesion proteins, which has been previously reported in the sequence of other fucoidanases like MfFcnA (Colin et al., 2006), Fhf1 (Vuillemin et al., 2020), and the three endo-fucoidanases, Fp273, Fp277, and Fp279 (Schultz-Johansen et al., 2018). The function of this domain in fucoidanases still remains unknown, but it is not considered involved directly in the catalytic activity of the GH107 enzymes (Vickers et al., 2018; Vuillemin et al., 2020). The second predicted domain is a 67 amino acids long secretion system T9SS C-terminal sorting domain (IPR026444), positioned from amino acid 842 to 909 in the C-terminal end of the Fhf2 sequence. This domain is present in other known fucoidanases, notably MfFcnA, FFA1, FFA2, AXE80_07420, and AXE80_07305 (Zueva et al., 2020) and most recently also found in Fhf1 (Vuillemin et al., 2020).

InterProScan was unable to predict the catalytic D1 domain in Fhf2, found in other known GH107 fucoidanases, since no domain number has been given yet (Figure 1; Vickers et al., 2018). Instead this was predicted by sequence alignments of Fhf2 and other known fucoidanases, using MfFcnA as template. The D1 domain was first identified in the crystal structures and consists of a (β/α)_8_-barrel (Vickers et al., 2018). The alignment suggesting that Fhf2 contains the conserved catalytic D1 domain between amino acids 24 and 426 (Supplementary Figure S1; Supplementary Table S1).

The D1 domain of Fhf2 from *F. haliotis* shares identity to the 20 closest fucoidanase sequences ranging from 20 to 88%. The highest identity was found with α(1,4) linkage specific FFA2 (88%), while lower identities were found with the other characterized endo-acting α(1,4) linkage specific fucoidanases, Fhf1 (63%), MfFcnA (60%), and FFA1 (59%). The D1 sequence of the fucoidanases Fda1 and Fda2, FcnA_19D and D1818_06655 from *Aquamarina* sp. BL5 showed the lowest identities with Fhf2 of 24, 24, 23, and 20%, respectively. The alignment showed that only seven residues in the catalytic D1 domain are conserved in all sequences (Fhf2: Y144, W170, D222, D227, G296, H297, and W355), although much higher conservation was found in the D1 domain between Fhf2 and the template MfFcnA sequence sharing 60% identity. D227 is predicted as the nucleophile and the H297 as the acid-base catalyst in Fhf2. The four residues that were present in the −1 subsite of MfFcnA are also identified in the Fhf2 sequence as Y144, N146, N270, and W355. Both the tyrosine and tryptophan (Y144 and W355 in Fhf2, respectively) are conserved in all GH107 enzymes investigated, while the asparagine (N146 in Fhf2) is changed to an alanine residue in Fda1 and Fda2, which are α(1,3) acting fucoidanases (Sakai et al., 2004) and to a serine residue in the uncharacterized fucoidanase D1818_06650.

### 3D Modeling of Fhf2

**Figure 2:**
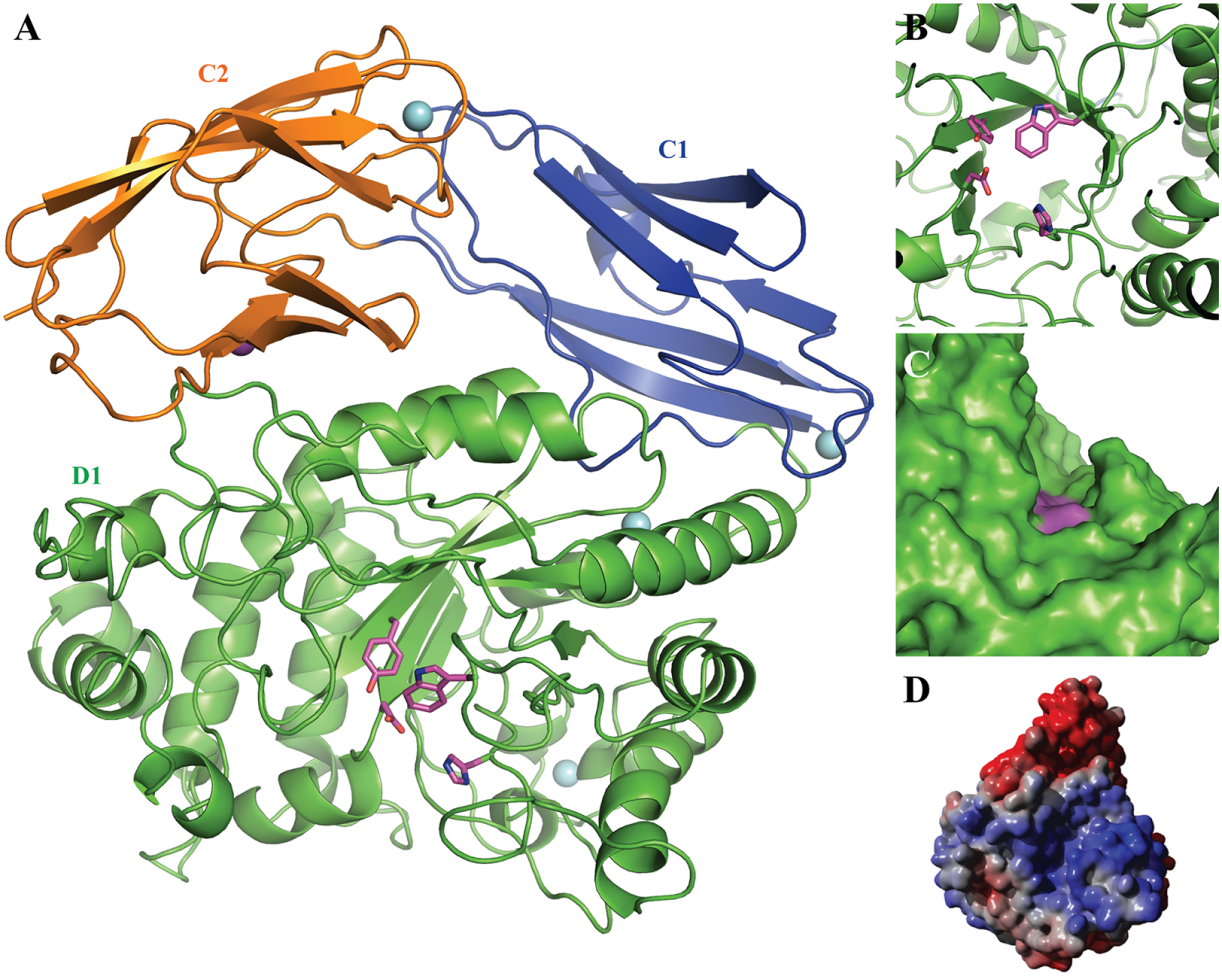
3D homology model of Fhf2. The putative N-terminal catalytic domain D1 is shown in green with the catalytic residues Asp227 and His297 as well as the conserved Tyr139 and Trp350 highlighted in pink; the C-terminal domains C1 and C2 are presented in blue and orange, respectively. The four predicted Ca^2+^ ions are indicated by cyan spheres and the predicted Na^+^ ion by a purple sphere. **(A)** The 3D model presented as cartoon, showing the beta-barrel fold of the predicted C1 and 2 domains as well as the beta-barrel fold surrounding the active site. **(B)** A zoom of the active site, nicely illustrating the active site amino acids. **(C)** A sphere model, showing the grove extending from the active site. **(D)** Electrostatic surface of the Fhf2 homology model: blue color indicates positively charged surface areas, while red color indicates negatively charged surface areas.

To investigate the Fhf2 enzyme further, a homology model was constructed (Figure 2). For quality evaluation of the 3D homology model of Fhf2, the full-length enzyme and a truncated variant (original residues 25–630) were first analyzed by QMEANDisCo (Waterhouse et al., 2018), YASARA Z-score analysis (Krieger and Vriend, 2014), and MolProbity (Chen et al., 2010). The QMEANDisCo Z-score was 0.55 for the full-length Fhf2 and 0.76 for the truncated model (the closer to 1.0, the better the model), and the YASARA Z-scores were −1.963 for the full-length model and −1.038 for the truncated variant (higher values indicate a better quality of the model). Similarly, the MolProbity scores were 1.22 for the full-length model and 0.98 for the truncated variant (here lower values indicate better quality).

The N-terminal catalytic domain showed the characteristic (β/α)_8_-barrel structure (Figure 2) as recently described for MfFcnA (Vickers et al., 2018) and furthermore supported the prediction of the D1 domain of Fhf2. Additionally, the homology model predicted two C-terminal domains (C1–C2), similar to the C-terminal domains in the template structure of the endo-fucoidanase MfFcnA4 (PDB:6DLH; Vickers et al., 2018). The local quality estimated by QMEANDisCo and the Ramachandran analysis from MolProbity identified a third C-terminal domain (original residues 631–910) before T9SS C-terminal sorting domain. The third domain was modeled as a disordered domain (not shown) and was mainly responsible for the lower quality of the full-length model. Furthermore, the Fhf2 homology model contained one predicted Na^+^ ion binding site and four predicted Ca^2+^ sites, including two calcium sites in the D1 domain, suggesting that the Fhf2 enzyme might be calcium-dependent, like other representatives of the GH107 family (Silchenko et al., 2017a,b; Vickers et al., 2018; Vuillemin et al., 2020). The active site of Fhf2 seem situated in an active site groove, which potentially could accommodate the positioning of a fucoidan molecule (Figures 2C,D). To support this hypothesis, a calculation of the electrostatic potential of the surface revealed a positively charged surface area in the active site groove (Figure 2D). This finding further substantiates the potential binding of the negatively charged fucoidan substrate in the active site. Interestingly, the non-catalytic domains C1 and C2, of unknown function, showed a completely negatively charged surface area.

### Activity of the C-Terminal Deletion Mutant Fhf2Δ484 on Fucoidan From *F. evanescens*

**Figure 3:**
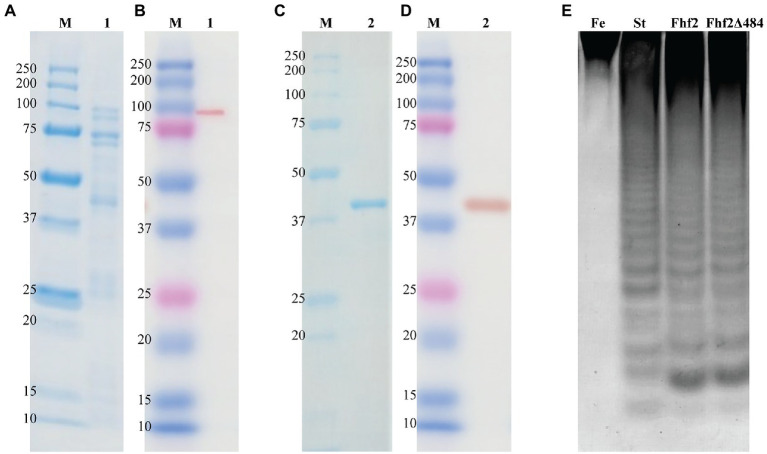
Purification and enzyme activity of Fhf2 and Fhf2Δ484. SDS-PAGE and Western blot of Fhf2 (**A,B** respectively) and Fhf2Δ484 (**C,D** respectively). (1) Fhf2 protein with the expected molecular weight of 98 kDa, including other protein bands with lower molecular weight, visible in the SDS-PAGE in **(A)**, but not in the western blot in **(B)**. (2) Purified Fhf2Δ484 protein with the expected molecular weight of 46 kDa and no other proteins visible. M) Protein marker. **(E)** Fucoidanase activity by C-PAGE of Fhf2 and Fhf2Δ484 on fucoidan fraction 2 extracted from *Fucus evanescens* (FeF2). (St) oligosaccharide products of the enzymatic reaction of FFA2 on fucoidan from *F. evanescens*. Reaction conditions were 0.9% substrate, 0.3 mg/ml enzyme, 10 mM Tris-HCl pH 7.4, 10 mM Ca^2+^, 125 mM NaCl, 35°C for 24 h.

Expression of the 98 kDa recombinant Fhf2 protein, lacking the predicted N-terminal signal peptide and the C-terminal secretion system sorting domain, was performed in *E. coli* with a C-terminal 6xHis-tag. The expression and purification of Fhf2 (Figure 3) resulted in a degraded protein giving several protein bands in the SDS-PAGE (Figure 3A), although only the protein at approximately 98 kDa gave a positive band in the western blot analysis (Figure 3B), suggesting degradation from the C-terminal end. In spite of the apparent degradation of the Fhf2 protein, it showed activity on fucoidan from *F. evanescens* as analyzed by Carbohydrate Polyacrylamide Gel Electrophoresis (C-PAGE; Figure 3E). C-PAGE is currently the main assay used for fucoidanase activity assessment, using small amounts of fucoidans.

The expression and robustness of GH107 enzymes have previously been improved by C-terminal truncations (Colin et al., 2006; Cao et al., 2018; Vuillemin et al., 2020). Hence, a severely truncated version of Fhf2, named Fhf2∆484 was constructed by removing a total of 484 amino acids from the C-terminal end of the native sequence, to improve enzyme expression and purification. The 484 amino acids of the truncated C-terminal included the InterProScan predicted T9SS C-terminal sorting domain and the cadherin-like superfamily domain (C1) as well as the 3D model-predicted C2 and C3 domains. The resulting Fhf2Δ484 protein was only 412 amino acids long and only contained the catalytic D1 domain with a predicted molecular weight of 46 kDa. The purified enzyme gave the expected band of 46 kDa (Figures 3C,D) and retained comparable activity to the full-length Fhf2 enzyme on fucoidan from *F. evanescens* (Figure 3E). Fhf2∆484 was selected for further characterization of the enzyme.

### Substrate Specificity of the Recombinant Fucoidanase Fhf2Δ484

**Figure 4:**
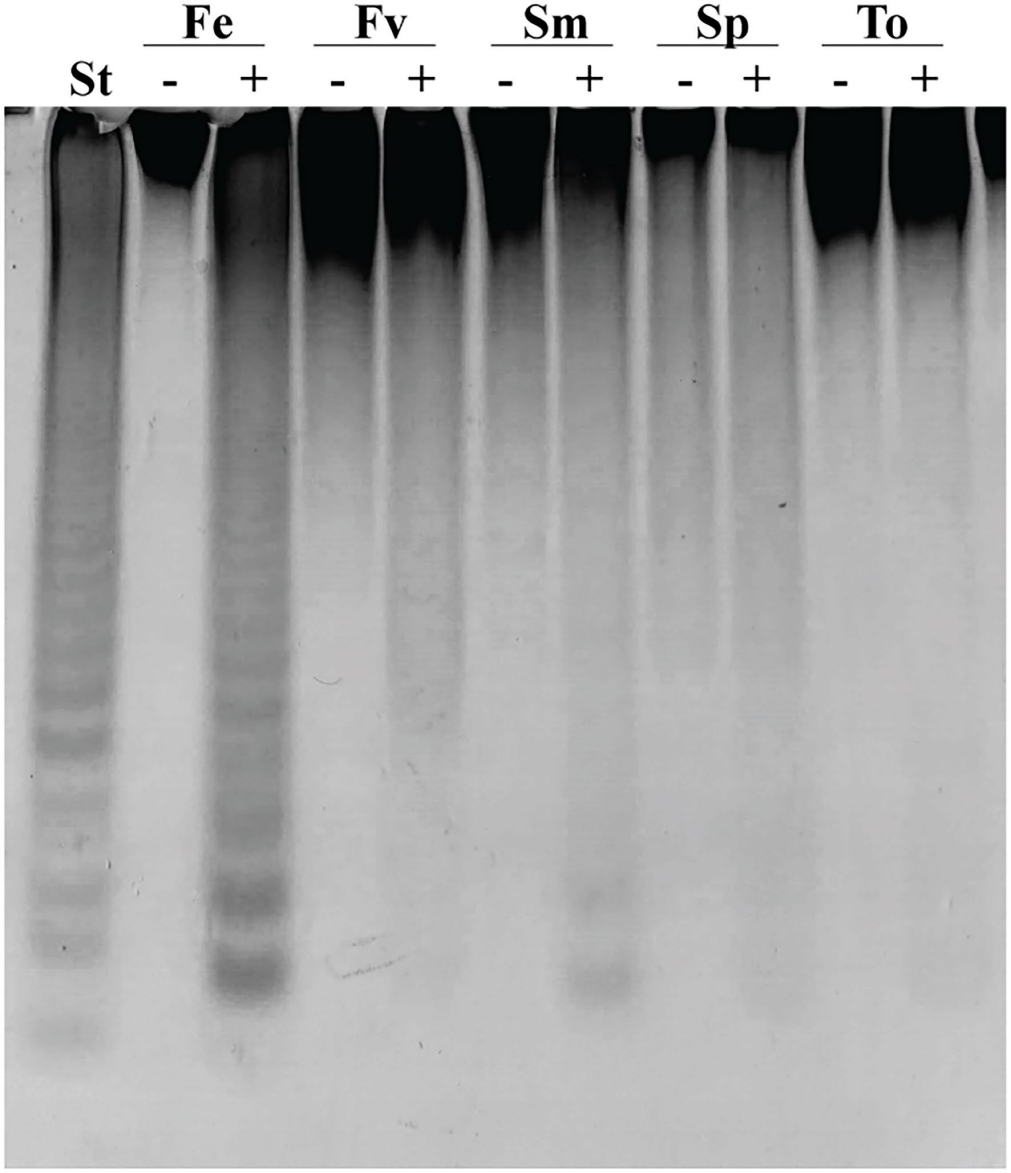
C-PAGE of Fhf2∆484 activity on fucoidans from different brown seaweed species. (−) indicates control substrate and (+) Fhf2∆484 enzymatic reactions on fucoidans from different seaweed species *F. evanescens* (Fe), *F. vesiculosus* (Fv)*, S. mcclurei* (Sm), *S. polycystum* (Sp) and *T. ornata* (To). (St) positive control reaction of FFA2 on *F. evanescens* fucoidan. Reaction conditions were 0.9% substrate, 0.3 mg/ml enzyme, 10 mM Tris-HCl pH 7.4 and 125 mM NaCl at 35°C for 24 h.

The substrate specificity of the fucoidanase Fhf2Δ484 was investigated by using six different fucoidan substrates, varying in their chemical fine-structures (Figure 4; Supplementary Figure S2). The products of the enzymatic reaction were analyzed by C-PAGE. The different bands visible in the C-PAGE gel represents different oligosaccharides and indicate endo-acting fucoidanase activity. The oligosaccharides move through the gel according to their size and amount of charges from the sulfate groups.

Fhf2Δ484 showed activity on all fucoidan substrates (Figure 4) except for *T. ornata* (Figure 4) and *S. latissima* (Supplementary Figure S2) fucoidans, which are composed of α(1,3)-bonded L-fucosyls and showed the highest activity on fucoidan from *F. evanescens* containing both α(1,3) and α(1,4) glycosidic bonds. Lower apparent activity was found on fucoidans from *F. vesiculosus* also containing α(1,3) and α(1,4) glycosidic bonds, although differently sulfated than *F. evanescens* fucoidan. Lower activity was also observed on the galactofucans from *S. mcclurei* and *S. polycystum*.

### Time-Dependent Activity of the Fhf2Δ484 Enzyme

**Figure 5:**
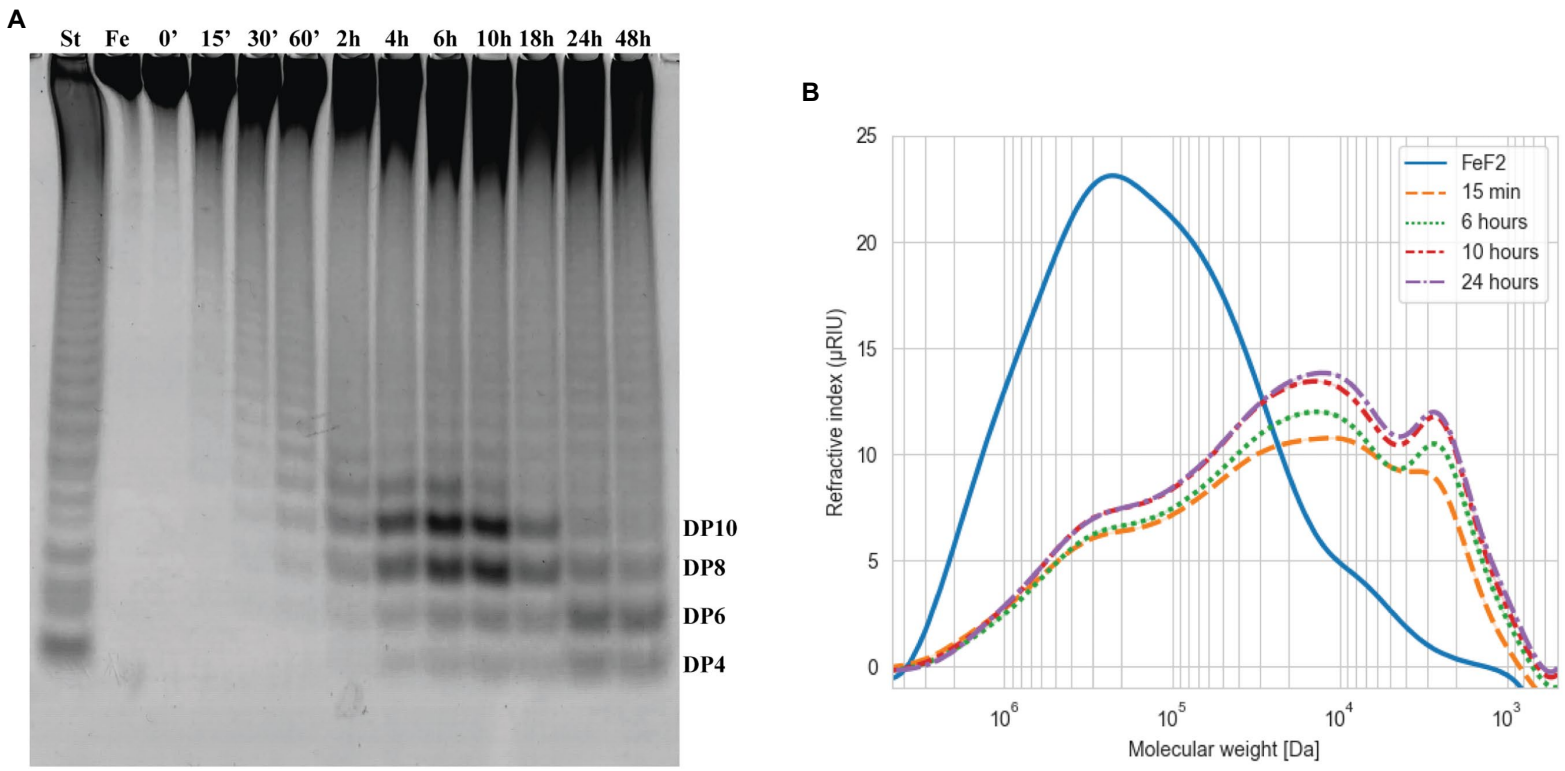
Time course of Fhf2∆484 fucoidanase on fucoidan from *F. evanescens*. The Fhf2∆484 activity was measured at different time points during 0–48 h of reaction and visualized by **(A)** C-PAGE or **(B)** HPSEC. (FeF2) fucoidan substrate from *F. evanescens*, (St) positive control, reaction of FFA2 on *F. evanescens* fucoidan. Tentatively suggested oligosaccharide sizes are indicated (DP4-10). Reaction conditions were 0.9% substrate, 0.3 mg/ml enzyme, 10 mM Tris-HCl pH 7.4, 125 mM NaCl at 35°C for varying reaction times. HPSEC data are shown in logarithmic scale and the molecular weight is in Da.

The fucoidanase Fhf2∆484 was assayed on fucoidan from *F. evanescens* from 0 to 48 h (Figure 5). The enzymatic activity was visible in the C-PAGE gel after 15 min of reaction, where substantial de-polymerization of the fucoidan substrate had already occurred, according to the HPSEC results (Figure 5). Longer reaction times released more oligosaccharides migrating as distinguishable bands in the C-PAGE gel. The oligosaccharide migrating furthest in the C-PAGE gel was detected after 4 h of reaction and co-migrated with the oligosaccharide migrating furthest of the standard. On a de-acetylated *F. evanescens* fucoidan standard, the band migrating furthest was previously determined as a fucose-tetra-saccharide (DP4) sulfated on C2 on all fucose residues (Silchenko et al., 2017b). The fucoidan from *F. evanescens* used here for the standard was not de-acetylated, so the suggestion that the band migrating the furthest is a tetra-saccharide is only tentative based on C-PAGE as the hydrolysis products here may contain acetylations, resulting in a slightly higher mass (structural data based on NMR analysis is discussed in the section Structure determination of the Fhf2∆484 fucoidanase hydrolysis products and mode of action of the enzyme, below). The third and fourth smallest oligosaccharide bands [likely octa (DP8) and decasaccharides (DP10)] were increasing in intensity from 4 to 10 h of reaction, before they were degraded to smaller sized oligosaccharides, as evident after 24 h of reaction. After 24 h of reaction, the enzyme had likely depleted the possible hydrolytic sites in the fucoidan and no further oligosaccharides were released after 48 h. Four hours was chosen for further experiments, to enable detection of more optimal conditions (e.g., release of more oligosaccharides), since the reaction at this time point was not completed. Together, the results strongly suggest that Fhf2 is an endo-acting fucoidanase enzyme.

### Optimal Conditions for Fhf2Δ484

**Figure 6:**
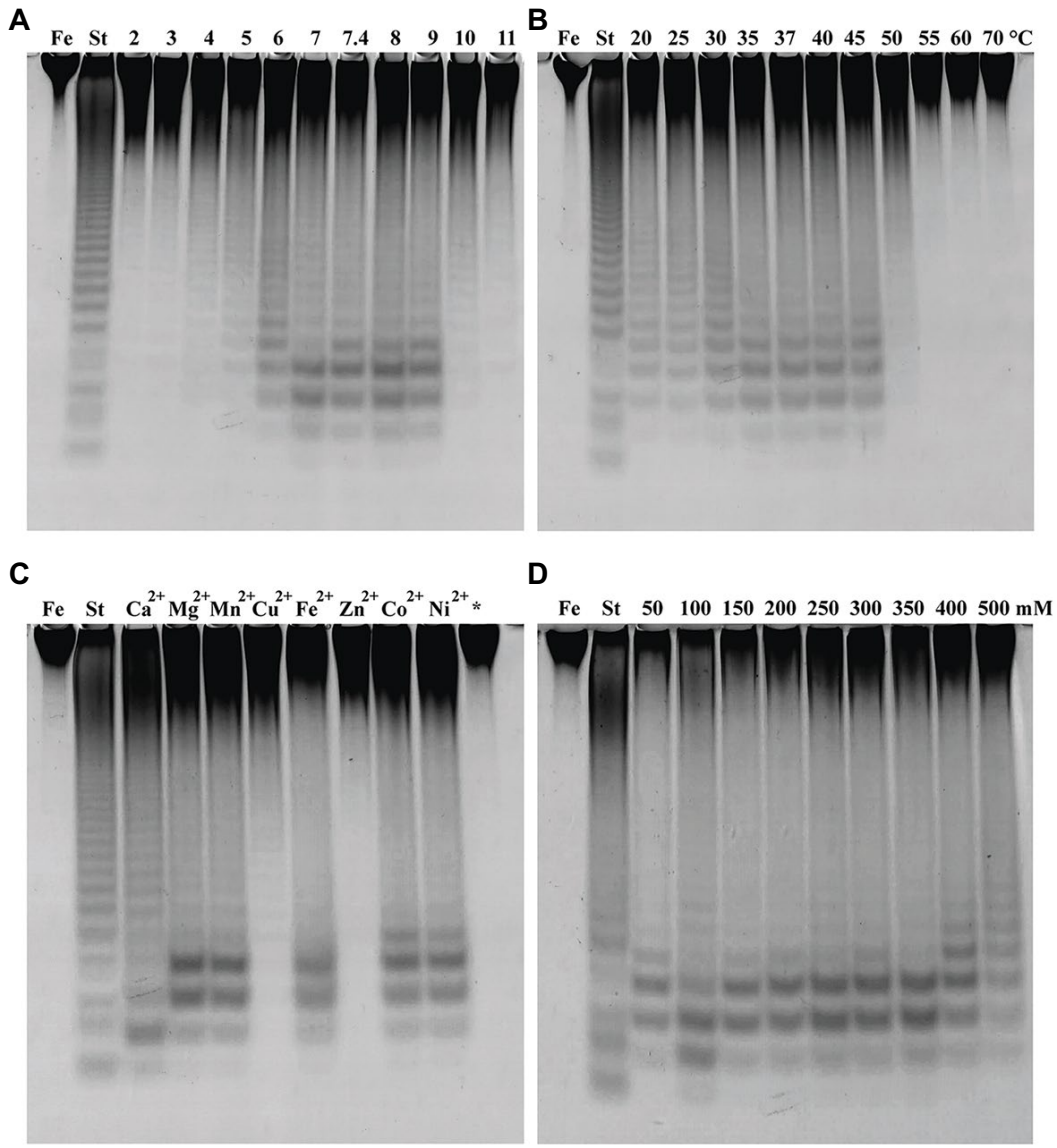
Fhf2∆484 activity under different reaction conditions shown by C-PAGE. Activity of Fhf2∆484 on fucoidan from *F. evanescens* (FeF2) under the influence of different **(A)** pH (at 35°C), **(B)** temperature (at pH 8), **(C)** divalent cations at 10 mM (pH 8 and 37°C), (*) indicates the activity after addition of EDTA, e.g., without presence of any divalent cations, and **(D)** NaCl concentration dependency (at pH 8 and 37°C). (St) positive control reaction of FFA2 on *F. evanescens* fucoidan. General reaction conditions were 0.9% substrate, 0.3 mg/ml enzyme, 10 mM Tris-HCl and 4 h reaction time.

The fucoidanase activity of Fhf2∆484 was measured at different pH on fucoidan from *F. evanescens* (Figure 6A). The enzyme showed activity from pH 4 to 10, with optimal activity at pH 8 to 9. At the strong acidic (pH 2, 3) and alkaline (pH 11) conditions, the enzyme was not active. pH 8 was chosen for further experiments.

The effect of temperature on Fhf2∆484 activity was measured at temperatures from 20 to 70°C on fucoidan from *F. evanescens* (Figure 6B). The enzyme showed activity at a wide range of temperatures from 20 to 50°C. The temperature optimum for the Fhf2∆484 fucoidanase was observed at 35–45°C. The enzyme was inactive at temperatures of 55°C and above. 37°C was chosen for further experiments. To evaluate the effects of different divalent cations on the activity of the fucoidanase Fhf2∆484, divalent cations were removed by treatment with the chelating agent ethylenediaminetetraacetic acid (EDTA), followed by addition of different divalent cations (Figure 6C).

Fhf2∆484 lost activity after EDTA treatment, suggesting that divalent cations are important for function. Addition of Zn^2+^ and Cu^2+^ re-activated Fhf2∆484, which is evident as a smear in the C-PAGE run. Distinct bands were visible when Cu^2+^ was added, indicating a higher re-activation with Cu^2+^ than Zn^2+^. Mg^2+^, Mn^2+^, Fe^2+^, Co^2+^, and Ni^2+^ re-activated Fhf2∆484 to a higher degree than Cu^2+^ and Zn^2+^, resulting in the release of higher amounts of fuco-oligosaccharide products, evident as clear and distinct bands. The highest re-activation of Fhf2∆484 was observed by the addition of Ca^2+^, evident by the production of the two furthest migrating fuco-oligosaccharides. These results indicate that Fhf2∆484 is a metal-dependent enzyme and that Ca^2+^ ions play an essential role in the catalytic activity of the fucoidanase. This interpretation was also supported by the 3D modeling of Fhf2, where two calcium-binding sites were predicted in the catalytic D1 domain (Figure 2).

The effect of NaCl concentrations on Fhf2∆484 activity was also investigated (Figure 6D). Optimal Fhf2∆484 activity was achieved at 100 mM NaCl, while the enzyme retained activity at all tested concentrations ranging from 50 to 500 mM, although the activity was slightly decreased at 50 and 500 mM NaCl. 100 mM NaCl was chosen for further analysis. Fhf2∆484 was only slightly affected by the NaCl concentration, comparable with results obtained for the fucoidanase Fhf1∆470. 100 mM NaCl was chosen for further analysis.

**Figure 7:**
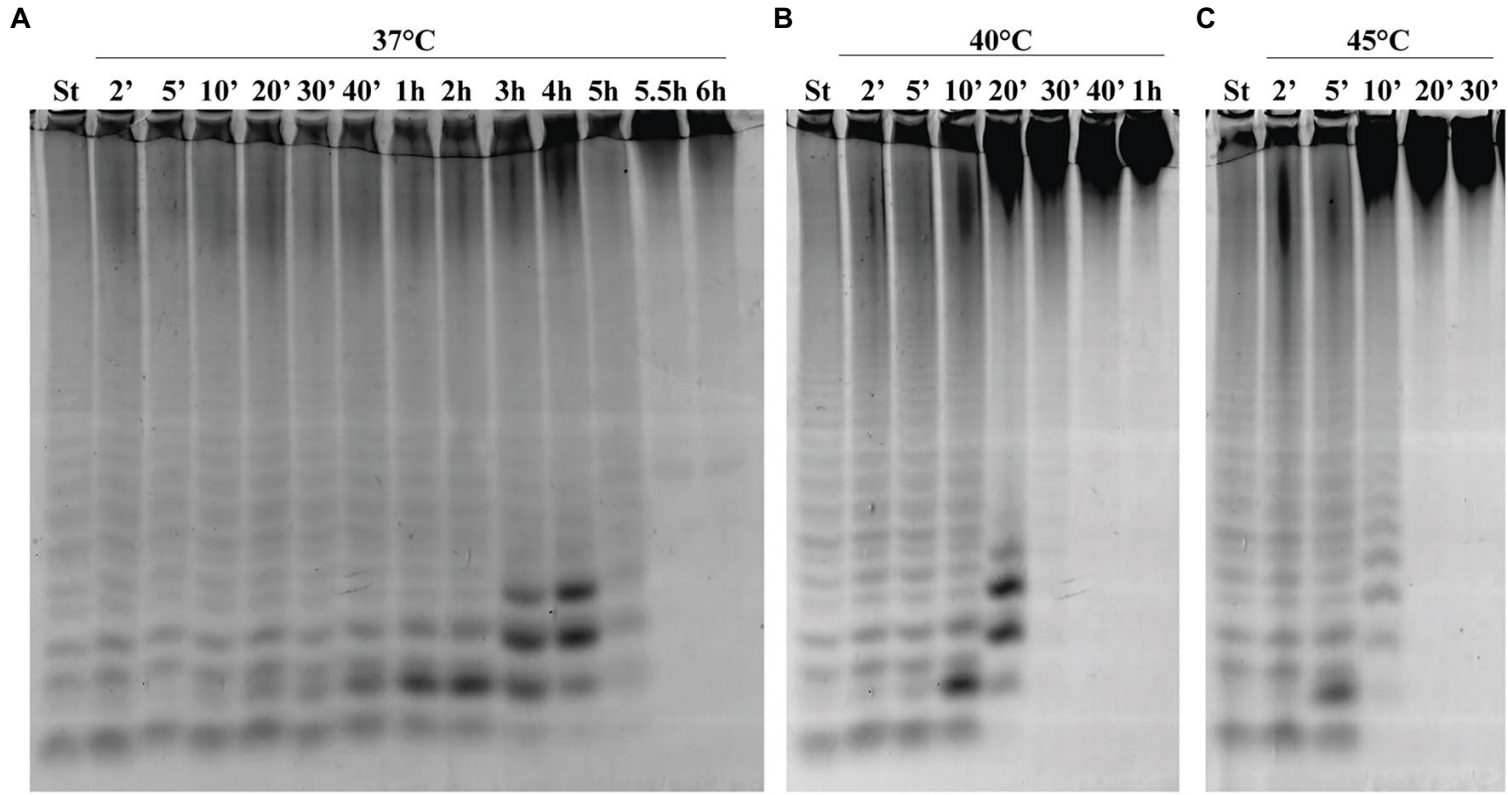
Thermostability of Fhf2∆484. Fhf2∆484 was incubated without substrate for the indicated time periods, before the activity was determined on fucoidan from *F. evanescens*. **(A)** 37°C, **(B)** 40°C, and **(C)** 45°C. (St) positive control reaction of FFA2 on *F. evanescens* fucoidan. Reaction conditions: 0.9% substrate, 0.3 mg/ml enzyme, 10 mM Tris-HCl pH 8, 100 mM NaCl, 37°C, 4 h reaction time.

To determine the thermostability of Fhf2∆484, the enzyme was incubated without substrate at different temperatures for different time periods before the activity was determined on fucoidan from *F. evanescens* (Figure 7). At 37°C, Fhf2∆484 retained maximum activity for 30 min, visible by the increase in the fuco-oligo-saccharide with suggested size of DP6. The activity slowly decreased from 40 min till 6 h, visible by the increase in the suggested DP6, 8 and 10, until 4 h, where after the release of all oligosaccharides severely decreased. At 40°C, the activity started decreasing at 10 min of incubation, while at 45°C the activity decreased already after 5 min of incubation (Figure 7). To validate the results further, the melting temperature of the enzyme was investigated in the absence of substrate and showed that Fhf2∆484 started aggregating at 43°C, while the melting temperature was determined to be 47.5 +/− 0.1°C, supporting the C-PAGE results.

### Structure Determination of the Fhf2∆484 Fucoidanase Hydrolysis Products and Mode of Action of the Enzyme

**Figure 8:**
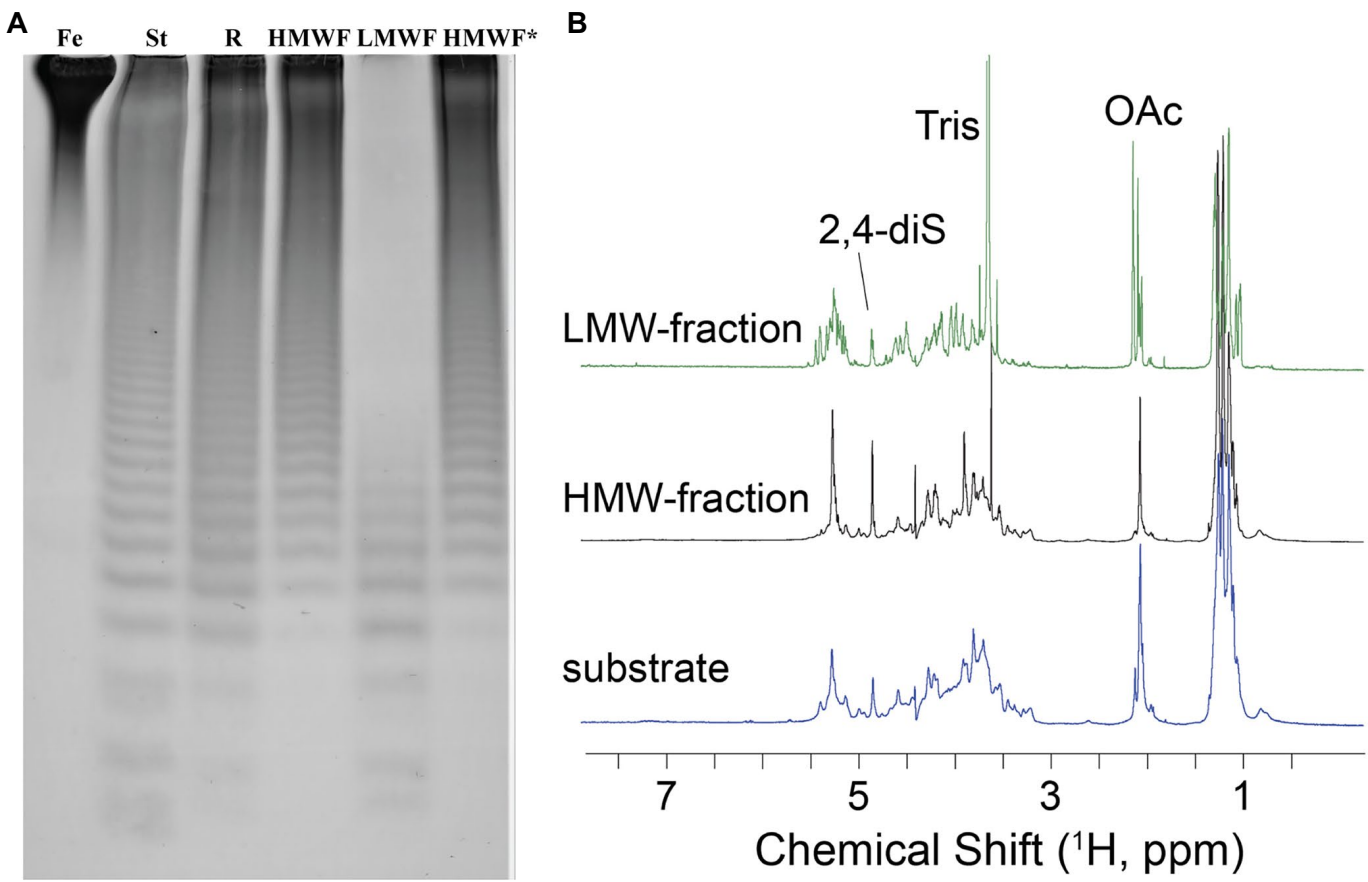
Characterization of the Fhf2∆484 hydrolysis products. **(A)** Enzymatic reaction of Fhf2Δ484 on fucoidan from *F. evanescens* shown by C-PAGE. (FeF2) control fucoidan substrate from *F. evanescens*. (R) Enzymatic reaction of Fhf2Δ484 on fucoidan from *F. evanescens*, (HMWF) high molecular weight fucoidan and (LMWF) low molecular weight fucoidan, (HMWF*) second enzyme treatment of HMWF with Fhf2Δ484. (St) positive control reaction of FFA2 on *F. evanescens* fucoidan. **(B)** ^1^H NMR spectra of *F. evanescens* fucoidan substrate (blue), high molecular weight fraction (black) and low molecular weight fraction (green) after hydrolysis by Fhf2Δ484.

To establish the detailed substrate specificity and the mode of action of Fhf2Δ484, the hydrolyzed products from the enzymatic reaction of Fhf2Δ484 on fucoidan from *F. evanescens* was separated into two fractions: low molecular weight fucoidan and high molecular weight fucoidan by ethanol precipitation and further investigated by NMR spectroscopy using one- and two-dimensional NMR-assignment spectra (^1^H, ^13^C, TOCSY, COSY, HMBC, and HSQC; Figure 8).

**Figure 9:**
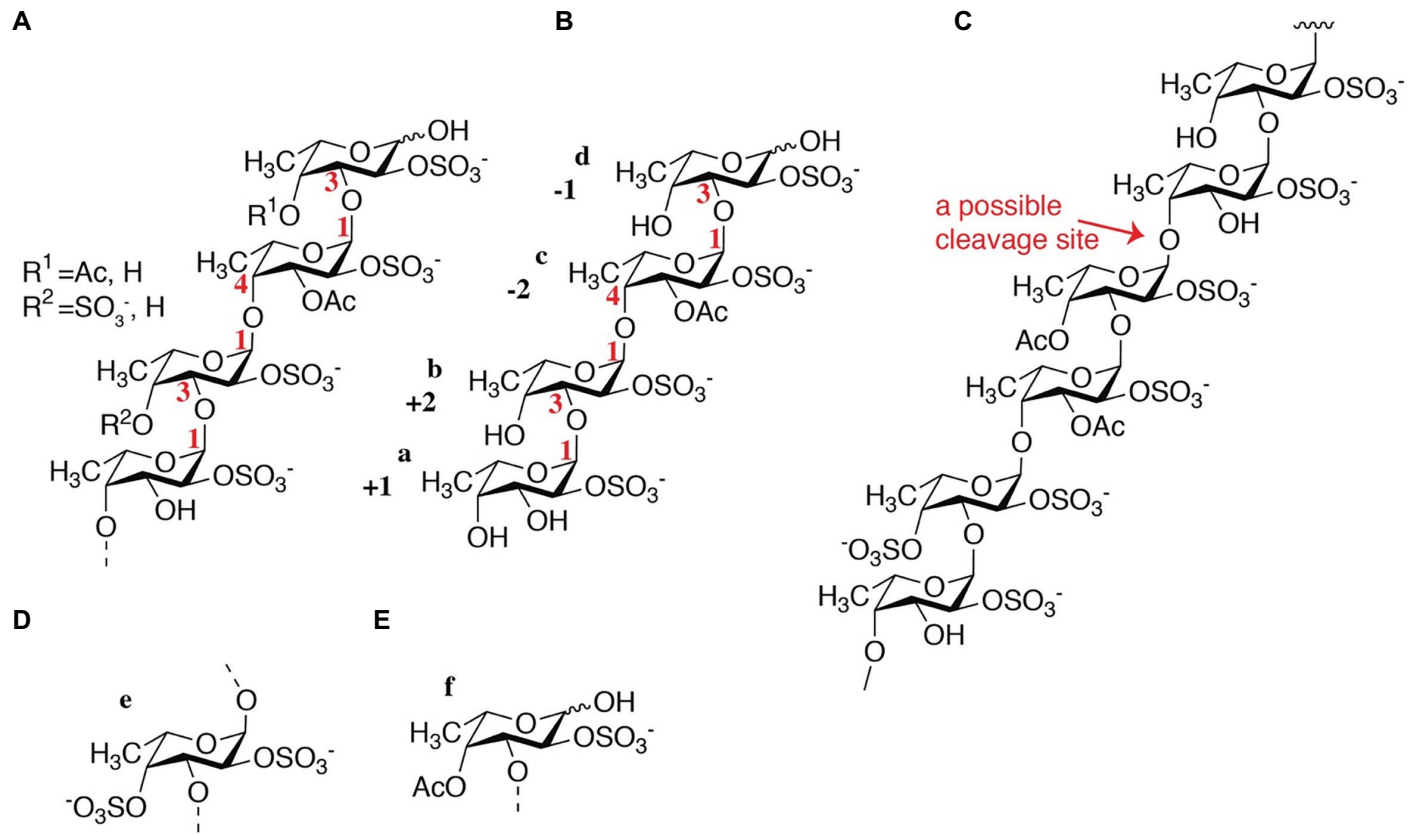
Chemical fine-structures of the LMWF oligosaccharides released by Fhf2Δ484 from fucoidan from *F. evanescens*. **(A)** General molecular structure containing 2-sulfated residues along 2-sulfated/3-acetylated, 2-sulfated/4-acetylated and 2-,4-disulfated residues; **(B)** Molecular structure of a main product; **(C)** Molecular structure of the substrate indicating the endo-1,4 cleavage site that can tolerate acetylated fucosyl units in the −1 position and 2,4-disulfated fucosyl units nearby; **(D)** 2,4-disulfated fucose occurs to some degree at non-terminal sites and **(E)** 2-sulfated and 4 acetylated fucose occurs to some degree at the reducing end.

According to the NMR spectroscopy analysis, the predominant molecular form of the low molecular weight fucoidan fraction was a sulfated tetra-saccharide with the following structure: α-L-Fucp2S-(1,3)-α-L-Fucp2S3Ac-(1,4)-α-L-Fucp2S-(1,3)-α-L-Fucp2S containing C2-sulfated residues (a, b, c) alongside C2-sulfated/C3-acetylated (c) residues. In addition, the oligosaccharide fraction contained reducing end C2-sulfated/C4-acetylated (f) residues and internal (non-terminal) C2,C4-disulfated residues (e; Figure 9). The chemical shifts of the low molecular weight fucoidans are summarized in Table 1. Previously, a similar oligosaccharide was released by fucoidanases FFA2 and Fhf1 on de-acetylated fucoidan from *F. evanescens* (Silchenko et al., 2017b; Vuillemin et al., 2020). The structure of the obtained low molecular weight fucoidan upon cleavage thus strongly indicates that Fhf2Δ484 is an endo-α(1,4)-acting fucoidanase that can tolerate 4-acetylation at the −1 site. Interestingly, 15% of C2,C4 disulfated residues were observed in the low molecular weight fucoidan fraction, in contrast to the products reported for FFA2 and Fhf1, where no C2,C4 disulfated residues were observed (Silchenko et al., 2014; Vuillemin et al., 2020). The C2,C4-disulfations found in the Fhf2Δ484 released low molecular weight fucoidan fraction was not in terminal residues, indicating that they were present internally most likely in longer oligosaccharides. These findings are significant, as it indicates that the Fhf2 enzyme may be a prospective novel candidate for producing oligosaccharides that are more highly sulfated than compounds produced with any previously described fucoidanases.

**Table 1:** The ^1^H and ^13^C NMR data for main structure (containing A, B, C, D units) oligosaccharide and variously substituted units (E, F) in the Fhf2Δ484-derived LMWF fraction (δ ^1^H/^13^C, ppm).

Residue		H1/C1	H2/C2	H3/C3	H4/C4	H5/C5	H6/C6
a	→4)-α-L-Fucp2OSO_3_^−^-(1→	5.33/95.6	4.66/73.8	4.37/68.5	3.91/73.4	4.58/68.9	1.25/16.6
b	→3)-α-L-Fucp2OSO_3_^−^-(1→	5.25/100.1	4.58/74.7	4.23/73.7	4.13/70.1	4.41/68.4	1.29/16.8
c	→4)-α-L-Fucp2OSO_3_^−^,3OAc-(1→	5.35/95.4	4.70/74.2	5.39/70.9	4.13/80.6	4.52/68.8	1.38/16.9
d^*^	→3)-α-L-Fucp2OSO_3_^−^-(1→	5.50/91.7	4.55/74.7	4.08/73.3	4.08/70.1	4.23/67.1	1.18/16.6
e	→3)-α-L-Fucp2,4OSO_3_^−^-(1→	5.35/99.7	4.60/74.8	4.31/73.2	4.95/79.9	4.50/68.0	1.32/17.3
f	→3)-α-L-Fucp2OSO_3_^−^,4OAc-(1→	5.54/91.8	4.61/75.0	4.26/n.d.	5.44/71.3	4.40/66.2	1.19/16.7

* α-Anomer.

**Figure 10:**
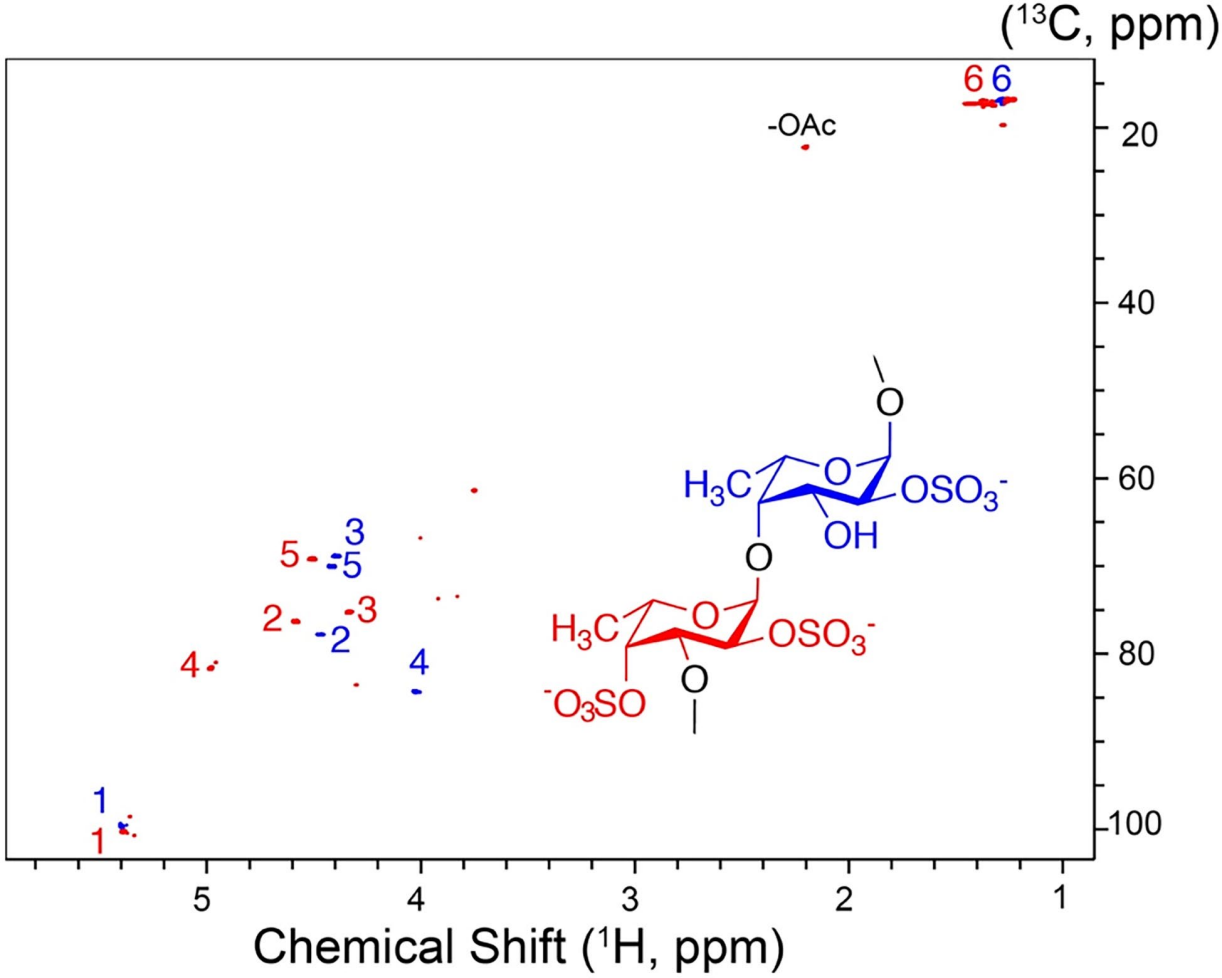
^1^H-^13^C NMR spectrum and molecular structure of the high molecular weight product obtained upon *F. evanescens* fucoidan degradation with Fhf2Δ484.

The high molecular weight fucoidan fraction yielded a homogeneous product as judged from the ^1^H-^13^C HSQC spectrum. Spectral analysis showed that the fraction mainly comprises repetitive polysaccharide consisting of alternating 2- and 2,4-disulfated fucose units linked as →3)-α-L-Fuc*p*2,4-di-S-(1,4)-α-L-Fuc*p*2S-(1→ (Figure 10). Spectrum and assignment resemble a regular fraction from *Fucus distichus* fucoidan (basionym *F. evanescens*; Bilan et al., 2004) of the identical structure.

By the data obtained, it is evident that the fucoidanase Fhf2Δ484 is able to catalyze the cleavage of α(1,4)-glycosidic bonds between 2O-sulfated L-fucose residues in the structural motif [→(3)-α-L-Fucp2S-(1,4)-α-L-Fucp2S-(1)→] of fucoidan isolated from *F. evanescens*.

### Determination of the Fhf2∆484 Fucoidanase Activity by FTIR

The α(1,4)-linkage specificity of Fhf2∆484 is similar to Fhf1∆470 on *F. evanescens* fucoidan (Vuillemin et al., 2020), comparative analysis was employed to estimate the enzymatic unit of Fhf2∆484 as previously determined for Fhf1∆470, FFA2, and MfFcnA using FTIR spectral fingerprinting (Tran et al., 2021, unpublished). In contrast to C-PAGE, which only requires very small amounts of fucoidan (~7 mg for all characterizations), FTIR demands quite large quantities of fucoidan (~2.5 g for all characterizations). For this reason, FTIR was only used for determination of the enzyme kinetics.

**Figure 11:**
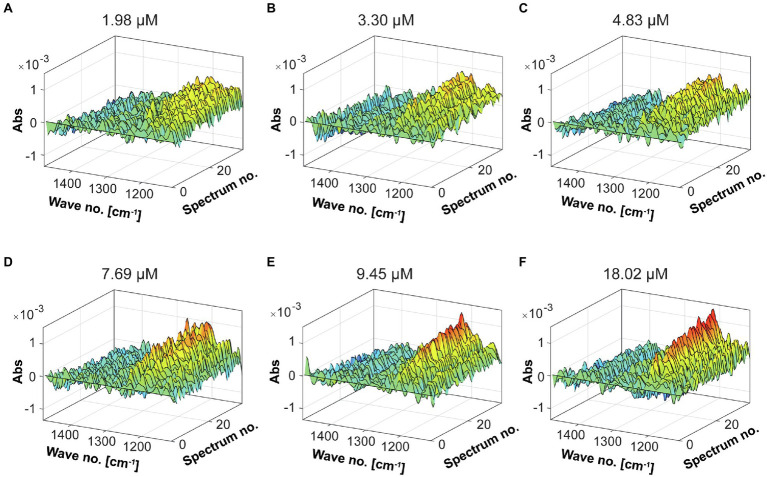
FTIR spectral evolution profiles for the Fhf2Δ484 endo-fucoidanase. The spectral evolution changes (30 spectra) in response to the enzyme concentration. The background spectra of buffer and substrate were subtracted. 2% w/v of fucoidans from *F. evanescens* was used at increasing enzyme dosages: **(A)** 1.98 μM, **(B)** 3.3 μM, **(C)** 4.83 μM, **(D)** 7.69 μM, **(E)** 9.45 μM, and **(F)** 18.02 μM.

To obtain the evolution profile during the enzymatic reaction, different enzyme dosages were used to evaluate the catalytic activity of the Fhf2∆484 fucoidanase by FTIR (Figure 11). Increased changes in the spectral evolution profiles were obtained with increasing enzyme dosages. The most notable increase in absorbance was observed in the range of 1,225–1,250 cm^−1^. This result was similar to the previously characterized enzymes, FFA2, FcnA2Δ229, and Fhf1Δ470, strongly suggesting that the FTIR evolution profile obtained reflects enzyme activity.

**Figure 12:**
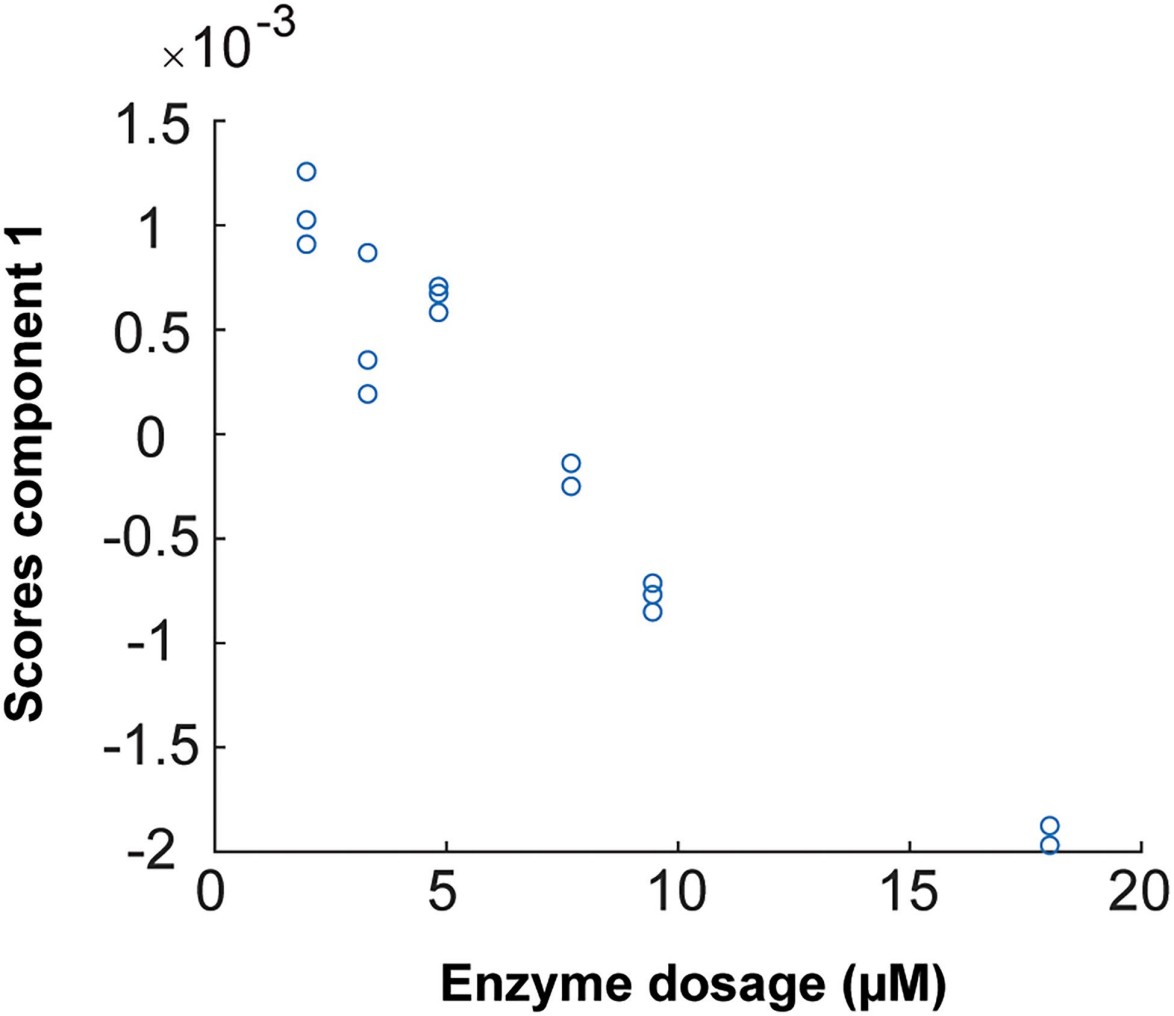
PARAFAC first component scores versus enzyme dosage plotted to build calibrations for Fhf2Δ484 on *F. evanescens*. The straight line, with the equation of *Y* = −0.0002 *X* + 0.0015, was fitted with the data. *R*^2^ = 0.95. Due to sign ambiguity in component analysis, the sign of the slope of the fitted calibration is not linked to substrate or product character.

The oscillations in the sulfate ester group after hydrolysis by the enzyme increase the concentration of the solute such that the optical absorbance of the solution decreases. This appears to be observed in the studied FTIR spectra. Therefore, combined with PARAFAC analysis (Figure 12), one enzymatic unit corresponds to the concentration of enzyme that is able to change the value of the scores by 0.01 (numeric value) during 498 s of reaction (8.3 min) on 20 g/L pure fucoidan from *F. evanescens* at 42°C, pH 7.4, 100 mM NaCl, and 10 mM CaCl_2_ (Tran et al., 2021, unpublished).

The linear equation results from the calibration curve of Fhf2∆484 action on fucoidan from *F. evanescens* (Figure 12) were Y = −0.0002 · X + 0.0015 (*R*^2^ = 0.95). Hence, the amount of Fhf2∆484 (concFhf2∆484) required to change the score value by 0.01 was 42.5 μM:


0.01=−0.0002⋅concFhf2Δ484+0.0015→concFhf2Δ484=42.5μM

And the specific activity of Fhf2Δ484 was measured to be 2.4 · 10^−4^ U*_f_*/μM.

## Discussion

In the present study, we identified the *Fhf2* gene from the genome of the marine bacteria *Formosa halioti*s. While the full-length enzyme was partially degraded during expression, we successfully expressed and purified the recombinant and C-terminally deleted fucoidanase Fhf2∆484. The successful expression of the truncated and active Fhf2∆484 was achieved by removal of 53% of the total amino acids from the C-terminal end of the native Fhf2. Several other fucoidanases require C-terminal truncation for successful purification (Colin et al., 2006; Cao et al., 2018; Vuillemin et al., 2020), while the current truncation of Fhf2∆484 is to date the largest described, where only the D1 catalytic domain remains. The two putative C-terminal domains, predicted by the 3D modeling and removed in Fhf2∆484 are likely not involved in the catalyzing function of the enzyme in *in vitro* experiments, although they might play a role in recognizing and/or binding to fucoidan substrates or binding to the seaweed surfaces under natural conditions, as has been seen for other polysaccharide specific T9SS domain containing enzymes (Veith et al., 2017).

Fhf2∆484 showed significantly higher temperature stability than previously characterized fucoidanases, exhibiting optimal activity at temperatures between 35 and 45°C and remains active after long pre-incubation times for up to 240 min at 37°C and up to 10 min at 40°C. The only fucoidanase with a similarly elevated temperature optimum from 38 to 45°C was found in the marine bacteria *Vibrio* sp. No-5 (Furukawa et al., 1992; Supplementary Table S2).

The functional characterization of Fhf2∆484 revealed that Fhf2 activity was affected by the addition of different divalent cations. This is consistent with previous reports on GH107 enzymes (Silchenko et al., 2017a,b; Vickers et al., 2018; Vuillemin et al., 2020; Supplementary Table S2), although the role of the metal ions on the activity of fucoidanases is mostly unexplored.

The substrate specificity of Fhf2∆484 has been investigated using different fucoidans isolated from various sources of brown algae showing structural differences, namely, *F. evanescens*, *F. vesiculosus*, *S. mcclurei*, *S. polycystum*, *S. latissima*, and *T. ornata*. Fhf2∆484 catalyzes most efficiently cleavage of α(1,4) glycosidic bonds in fucoidan from *F. evanescens*. The activity of Fhf2∆484 seems to be influenced by the sulfation pattern/degree of fucoidan substrates, since the fucoidan from *F. vesiculosus* with higher sulfation degree, is not as efficiently degraded by Fhf2∆484, while the backbone structure and linkages are presumably the same. This difference in fucoidanase activity between differently sulfated fucoidan substrates has been observed for other previously characterized fucoidanases like Fhf1 (Cao et al., 2018; Vuillemin et al., 2020). Unlike Fhf1Δ470, Fhf2∆484 showed hydrolytic activity not only on fucoidan with a simple backbone structure from *F. evanescens* and *F. vesiculosus*, but also on complicated galactofucans from *S. mcclurei* and *S. polycystum*.

Since Fhf1 and Fhf2 both originate from *F. haliotis*, the biological relevance of the two enzymes might be reflected in the evident differences in substrate specificity and enzyme affinity. While Fhf1 (Fhf1Δ470) appears to catalyze fast hydrolysis having a specific activity of 1.2 · 10^−3^ U*_f_*/μM as measured by FTIR on 2% w/v *F. evanescens fucoidan* at 42°C, pH 7.4, 100 mM NaCl, and 10 mM CaCl_2_ (Tran et al., 2021, unpublished), the Fhf2 enzyme (Fhf2∆484) works comparably slower, having a specific activity of 2.4 · 10^−4^ U*_f_*/μM, but has a more promiscuous substrate selectivity and has a broader temperature span. This difference in activity and selectivity could enable the bacterium to degrade a wide range of fucoidans. Compared to other characterized fucoidanases, Fhf2∆484 appears to be able to act effectively over wide ranges of pH, temperature, salt concentration and on a wide range of substrates, thus compounding properties that are useful for industrial purposes.

Furthermore, the ability to produce homogenous fucoidan oligosaccharides from the very heterogeneous native *F. evanescens* fucoidan is a very valuable ability in an industrial point of view. An interesting feature of Fhf2*Δ*484 the production of oligosaccharides with slightly higher molecular weight, likely octa and decasaccharides if the reaction is stopped before completion, when compared to other fucoidanases that hydrolyze to tetra-saccharides immediately (Silchenko et al., 2017b; Vuillemin et al., 2020). The very low molecular weight of the tetra-saccharides could potentially lead to reduction in bioactivity, compared to slightly higher molecular weight fucoidans (Yang et al., 2008; Cho et al., 2011). Hence, the octa and decasaccharides produced by hydrolysis of *F. evanescens* fucoidan by Fhf2*Δ*484 are unique and promising for the production of bioactive oligosaccharides. Finally, higher sulfation degree has previously been implicated in higher bioactivity (Cho et al., 2011; Ohmes et al., 2020) and the ability of Fhf2*Δ*484 to produce homogenous fucoidan oligosaccharides with higher degree of sulfation, releasing substantial amounts of 2,4-disulfated fucose containing oligosaccharides, might show interesting bioactive features.

## Conclusion

From the genome of *F. haliotis*, the endo-fucoidanase-encoding gene *fhf2* was identified. Stabilization of the Fhf2 enzyme by C-terminal truncation resulted in successful expression and purification of the Fhf2∆484 enzyme. Fhf2∆484 hydrolyze α(1,4) fucosyl linkages with C2 sulfations, but allows 2,4-disulfations in longer oligosaccharides in contrast to Fhf1. Fhf2∆484 releases higher molecular weight fuco-oligosaccharides, likely octa- and decasaccharides, unlike other fucoidanases that release oligosaccharides of all sizes at comparable amounts. Fhf2∆484 exerts activity on an array of different fucoidan substrates from brown seaweeds, even the very complex *S. polycystum* and *S. mcclurei* galactofucans. It shows activity for an extended time at slightly higher temperatures than previously characterized fucoidanases. Taken together, the results provided here suggest that Fhf2∆484 shows potential for the production of fuco-oligosaccharides for in-depth elucidation of fucoidan structures from different seaweed species and bioactivity assessments with regard to the different size obtained and the different sulfation degree.

## Data Availability Statement

The data sets presented in this study can be found in online repositories. The names of the repository/repositories and accession number(s) can be found in the article/Supplementary Material.

## Author Contributions

MM, VoT, and AM: conceptualization. VoT, MM, MV, SM, HC, JM, TN, VyT, and JH: experiments and analytical work. VoT, MM, SM, VP, and AM: data interpretation. MM, TV, HK, and AM: supervision and funding. VoT and MM: original draft preparation. VoT, MM, and AM: manuscript writing and editing. All authors contributed to the article and approved the submitted version.

## Funding

This project was funded by MARIKAT JPI Cofund Blue BioEconomy Project grant number 9082-00021B and by the Technical University of Denmark. MC and TR acknowledge support from ANR under grant ANR-10-BTBR-04 (investment expenditure program IDEALG). This research was furthermore funded by Vietnam National Foundation for Science and Technology Development (NAFOSTED) under Grant number 106.02-2018.353, and this study was part of the FucoSan-Health from the Sea Project, supported by EU InterReg-Deutschland-Denmark and the European Fund of Regional Development. NMR spectra were recorded at the NMR Center DTU, supported by the Villum Foundation.

## Conflict of Interest

The authors declare that the research was conducted in the absence of any commercial or financial relationships that could be construed as a potential conflict of interest.

## Publisher’s Note

All claims expressed in this article are solely those of the authors and do not necessarily represent those of their affiliated organizations, or those of the publisher, the editors and the reviewers. Any product that may be evaluated in this article, or claim that may be made by its manufacturer, is not guaranteed or endorsed by the publisher.

## Footnotes

^1^ http://www.cbs.dtu.dk/services/SignalP/

^2^ https://www.ebi.ac.uk/interpro/

^3^ https://www.ebi.ac.uk/interpro/search/sequence/
