# Supplementary material for: The Endo-α(1,4) Specific Fucoidanase Fhf2 From Formosa haliotis Releases Highly Sulfated Fucoidan Oligosaccharides
Source: Front Plant Sci. 2022 Feb 2;13:823668. doi: 10.3389/fpls.2022.823668 (PMC8847386; doi:10.3389/fpls.2022.823668)
Supplement: Supplementary file 1 [file Table_1.DOCX]

**Supplementary Material**

**The endo-α(1,4) specific fucoidanase Fhf2 from *Formosa haliotis* releases highly sulfated fucoidan oligosaccharides**

Vo Thi Dieu Trang^1,2,#^, Maria Dalgaard Mikkelsen^1.#^, Marlene Vuillemin^1^, Sebastian Meier^3^, Hang Thi Thuy Cao^1,2^, Jan Muschiol^1,4^, Valentina Perna^1^, Thuan Thi Nguyen^1,2^, Vy Ha Nguyen Tran^1,2^, Jesper Holck^1^, Tran Thi Thanh Van^2^, Huynh Hoang Nhu Khanh^2^ and Anne S. Meyer^1*^

*^1^Protein Chemistry and Enzyme Technology Section, Dept. of Biotechnology and Biomedicine, Technical*

*University of Denmark, 2800 Kgs Lyngby, Denmark*

*^2^NhaTrang Institute of Technology Research and Application, Vietnam Academy of Science and Technology, 02 Hung Vuong Street, Nhatrang 650000, Socialist Republic of Vietnam*

*^3^ Department of Chemistry, Technical University of Denmark, 2800 Kgs Lyngby, Denmark*

*^4^GEOMAR Helmholtz Centre for Ocean Research Kiel, Düsternbrooker Weg 20, 24105 Kiel, Germany*

^#^ These authors contributed equally

^*^Corresponding author, address as above, e-mail: asme@dtu.dk

**Table S1** Sequence identity of the N-terminal catalytic D1 domain of Fhf2 and 20 other fucoidanases. The percentage of identity and query coverage between the sequences have been performed by BLASTp tool.

| Fucoidanase | Organism | Accession number | Specificity | Length of D1 domain | Query Coverage (%) | Identity (%) |
| --- | --- | --- | --- | --- | --- | --- |
| FFA2 | *F. algae* KMM 3553T | WP057784219.1 | α(1,4) | 436 | 99 | 88.03 |
| Fhf1 | *F. haliotis* | WP066217780 | α(1,4) | 436 | 100 | 62.90 |
| MfFcnA | [*M. fucanivorans*](http://www.ncbi.nlm.nih.gov/Taxonomy/Browser/wwwtax.cgi?id=264023) SW5 | [CAI47003.1](http://www.ncbi.nlm.nih.gov/entrez/viewer.fcgi?db=protein&val=CAI47003.1) | α(1,4) | 421 | 98 | 59.86 |
| FFA1 | *F. algae* KMM 3553T | WP057784217.1 | α(1,4) | 418 | 100 | 59.25 |
| AXE80_07425 | [*W. fucanilytica* CZ1127](http://www.cazy.org/b6448.html) | [ANW96116.1](http://www.ncbi.nlm.nih.gov/entrez/viewer.fcgi?db=protein&val=ANW96116.1) | *nd* | 424 | 99 | 56.57 |
| D1818_06650 | *Aquamarina sp.* BL5 | AXT50524.1 | *nd* | 394 | 09 | 54.55 |
| AXE80_07305 | [*W. fucanilytica* CZ1127](http://www.cazy.org/b6448.html) | [ANW96097.1](http://www.ncbi.nlm.nih.gov/entrez/viewer.fcgi?db=protein&val=ANW96097.1) | *nd* | 426 | 95 | 47.90 |
| Fp279 | Uncultured bacterium | AYC81240.1 | *nd* | 452 | 92 | 44.69 |
| AXE80_07310 | [*W. fucanilytica* CZ1127](http://www.cazy.org/b6448.html) | [ANW96098.1](http://www.ncbi.nlm.nih.gov/entrez/viewer.fcgi?db=protein&val=ANW96098.1) | *nd* | 682 | 95 | 42.51 |
| Fp277 | Uncultured bacterium | AYC81239.1 | *nd* | 438 | 99 | 42.44 |
| AXE80_07420 | [*W. fucanilytica* CZ1127](http://www.cazy.org/b6448.html) | [ANW96115.1](http://www.ncbi.nlm.nih.gov/entrez/viewer.fcgi?db=protein&val=ANW96115.1) | *nd* | 592 | 92 | 35.45 |
| Fp273 | Uncultured bacterium | AYC81238.1 | *nd* | 620 | 84 | 35.23 |
| FcnA_5A | *Psychromonas sp*. SW5A | AYF59291.1 | *nd* | 388 | 07 | 34.38 |
| FNB79_00785 | *F. sediminum* PS13 | QDO92576.1 | *nd* | 389 | 06 | 32.14 |
| Fleli_2704 | *B. litoralis* DSM6794 | AFM05060.1 | *nd* | 393 | 17 | 28.00 |
| SVI_0379 | [*S. violacea* DSS12](http://www.cazy.org/b1202.html) | [BAJ00350.1](http://www.ncbi.nlm.nih.gov/entrez/viewer.fcgi?db=protein&val=BAJ00350.1) | *nd* | 352 | 67 | 26.64 |
| Fda1 | [*Alteromonas* sp. SN-1009](http://www.ncbi.nlm.nih.gov/Taxonomy/Browser/wwwtax.cgi?id=232) | [AAO00508.1](http://www.ncbi.nlm.nih.gov/entrez/viewer.fcgi?db=protein&val=AAO00508.1) | α(1,3) | 427 | 82 | 24.39 |
| Fda2 | [*Alteromonas* sp. SN-1009](http://www.ncbi.nlm.nih.gov/Taxonomy/Browser/wwwtax.cgi?id=232) | [AAO00509.1](http://www.ncbi.nlm.nih.gov/entrez/viewer.fcgi?db=protein&val=AAO00509.1) | α(1,3) | 494 | 82 | 23.92 |
| FcnA_19D | *Psychromonas sp*. SW19D | AYF59292.1 | *nd* | 388 | 14 | 22.95 |
| D1818_06655 | *Aquamarina sp.* BL5 | AXT50525.1 | *nd* | 391 | 30 | 20.00 |

**Table 2** Characteristics of fucoidanases comparing data from this study with available literature data.

| **Bacteria** | **Fucoidanase** | **kDa** | **Cleaves bond** | **Temp. interval (optimum)** | **pH interval (optimum)** | **Divalent cations that inhibit** | **Divalent cations that activate** | **NaCl con. interval (optimum)** | **Reference** |
| --- | --- | --- | --- | --- | --- | --- | --- | --- | --- |
| *Formosa haliotis* | Fhf2 | 98 |  | 35-45 | 8 | Cu^2+^, Zn^2+^ | Ca^2+^_,_ Mg^2+^, Mn^2+^, Fe^2+^, Co^2+^ Ni^2+^ | 100mM | *This study* |
|  | Fhf1 | 121 | α(1,4) | 37-40 | 8 | Mg^2+^ Cu^2+^, Fe^2+^ | Ca^2+^, Mn^2+^, Zn^2+^, Ni^2+^, | 100mM | (Vuillemin et al., 2020) |
| *Mariniflxile fucanivorans* SW5 | MfFcnA | 105 | α(1,4) | 20-25 | 7.5 | Nd | Ca^2+^ | Nd | (Descamps et al., 2006) |
| *Formosa algae* KMM 3553^T^ | FFA1 | 96 | α(1,4) | 37^(*)^ | 6.5-9.1 | Cu^2+^, Zn^2+^ | Ca^2+^, Mg^2+^, Ba^2+^ | Nd | (Silchenko et al., 2017a) |
|  | FFA2 | 101 | α(1,4) | 25-37 | 6.5-9.1 | Al^2+^, Cu^2+^, Sn^2+^, Fe^3+^ | Ca^2+^, Mg^2+^, Ba^2+^, Co^2+^, Mn^2+^ | Nd | (Silchenko et al., 2017b) |
| *Vibrio sp.* No5 | - | 68 | exo | 38-45 | 6 | Hg^2+^, Fe^3+^, Ag^+^ | Co^2+^ | Nd | (Furukawa et al., 1992) |
| *Alteromanas sp.* SN-1009 | Fda1 and Fda2 | 100/94 | α(1,3) | 30-35 | 6.5-8 | Cu^2+^, Zn^2+^ | Ca^2+^ | 400mM | (Sakai et al., 2004) |
| *Wenyingzhuangia fucanilytica CZ1127^T^* | FWf1 | 89 | α(1,4) | 24-35 | 6.4-7.2 | Al^3+^, Co^2+^, Cu^2+^, Fe3^+^, Mn^2+^, Pb^2+^, Sn^2+^, Ni^2+^ | Ca^2+^ | 100-300mM | (Zueva et al., 2020a) |
|  | FWf2 | 98 | α(1,4) | 24-40 | 6.0-6.8 | Al^3+^, Co^2+^, Cu^2+^, Fe3^+^, Mn^2+^, Pb^2+^, Sn^2+^, Ni^2+^ | Ca^2+^ | 100-300mM |  |

Nd: Not determined

*: The assay condition, not investigate optimum.

**Figures** **(legend for Figure S1 on p. 8)**


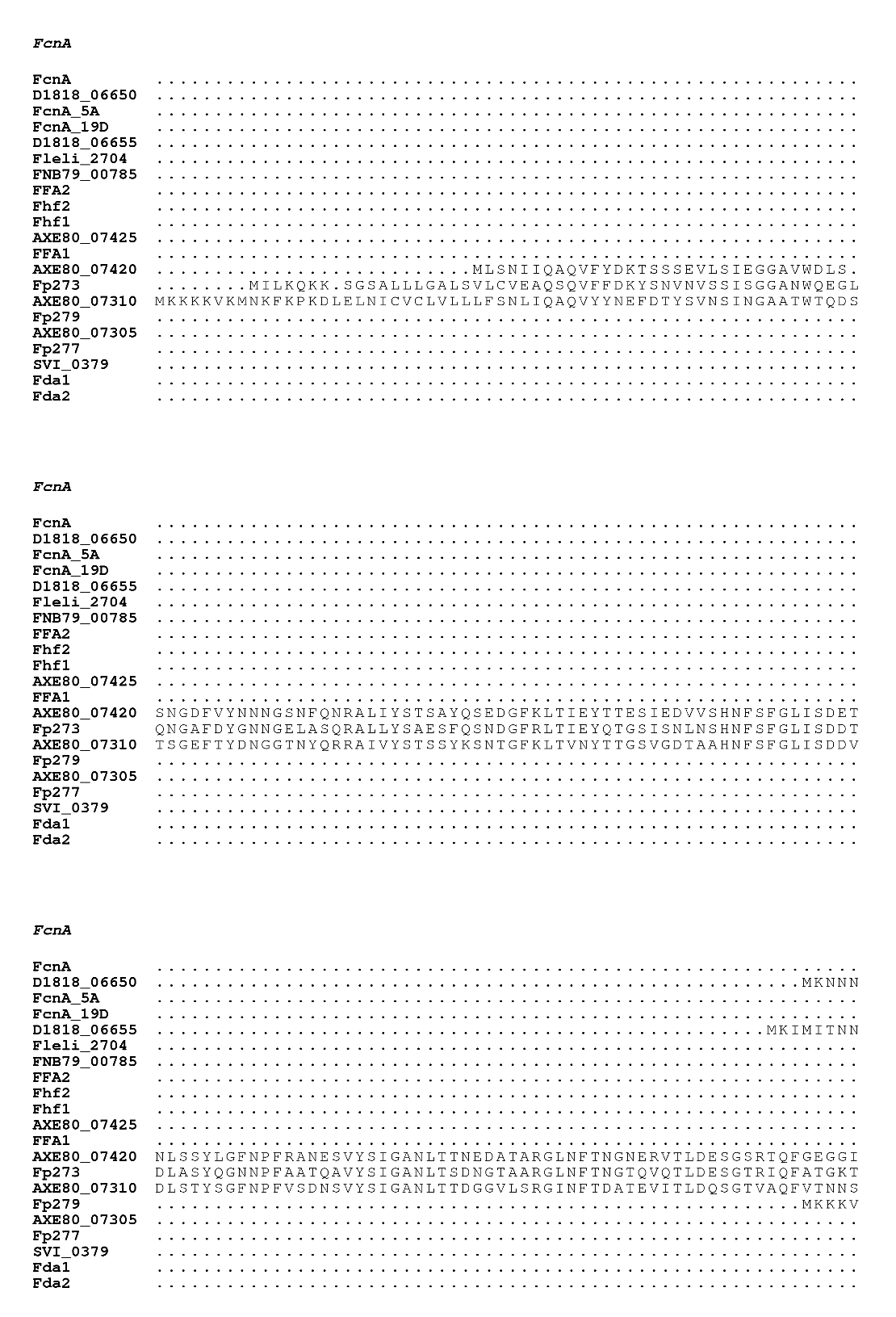


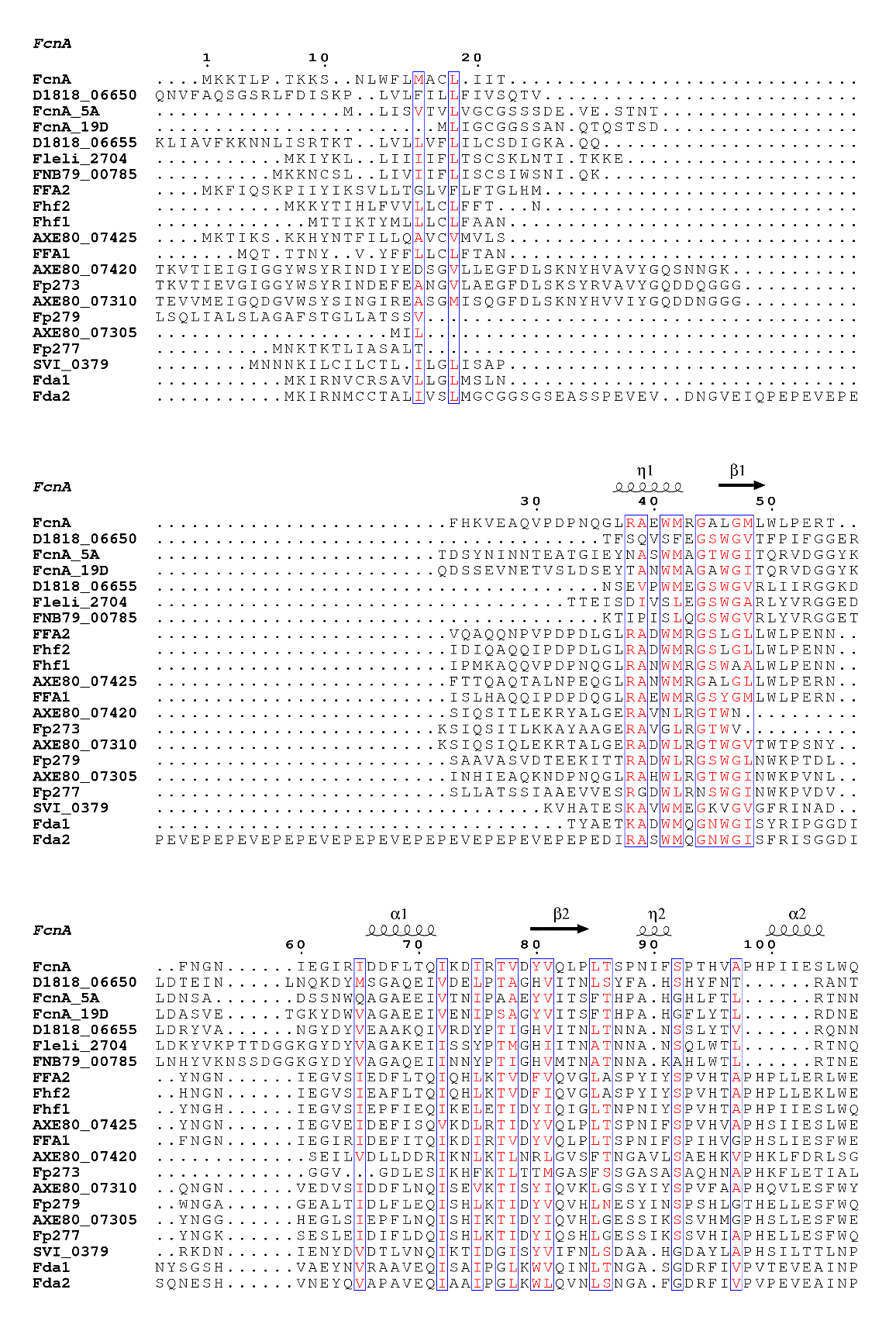

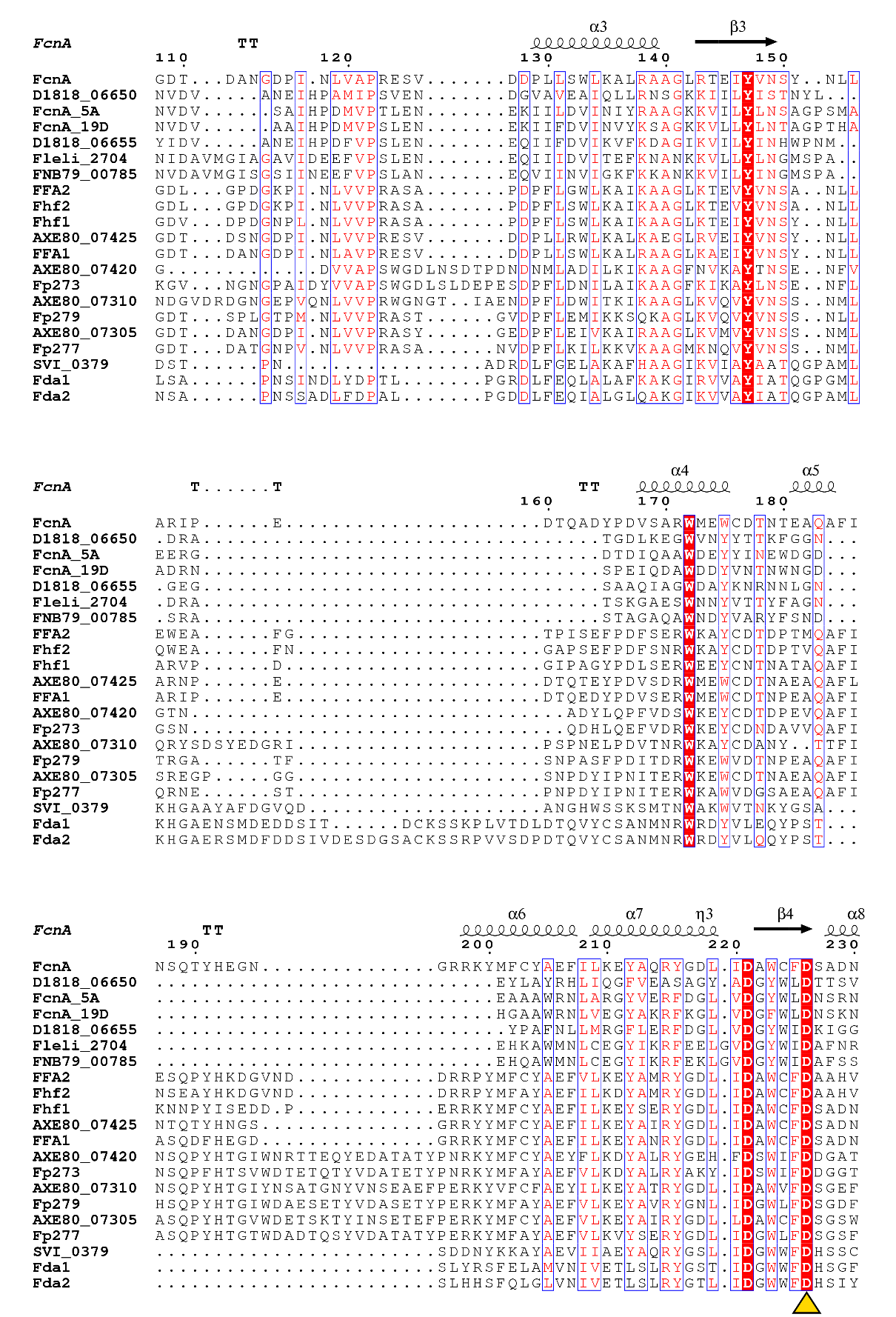

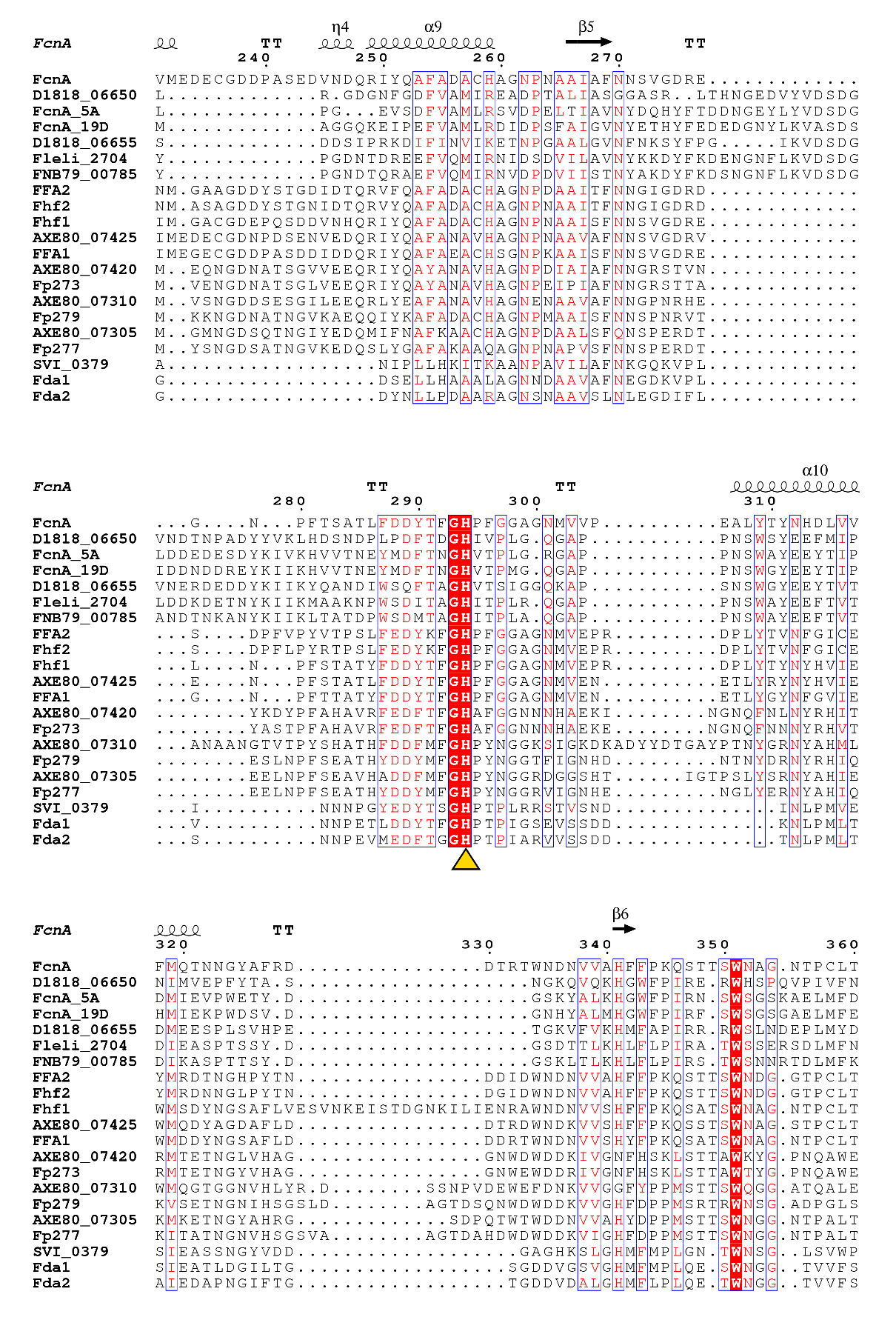

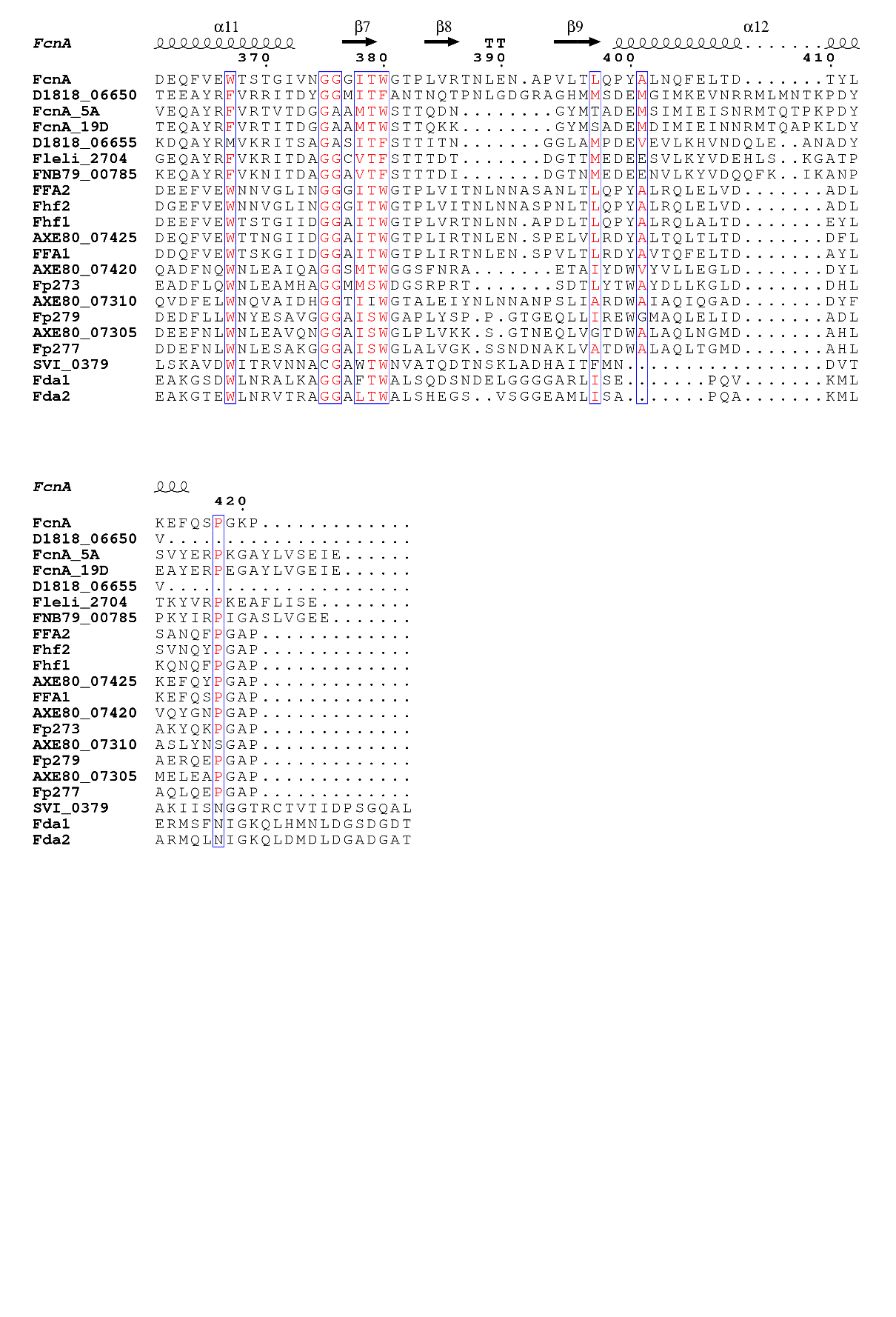


**Figure S1. Multiple alignments of D1 domain sequences of fucoidanases.** The multiple alignments were made from D1 domain of 21 sequences, including Fhf2 (accession number WP066217784.1) and Fhf1 (WP066217780.1); FFA1 (WP057784217.1) and FFA2 (WP057784219.1)*;* FcnA ([CAI47003.1](http://www.ncbi.nlm.nih.gov/entrez/viewer.fcgi?db=protein&val=CAI47003.1))*;* FcnA_5A (AYF59291.1); FcnA_19D (AYF59292.1); AXE80_07420 ([ANW96115.1](http://www.ncbi.nlm.nih.gov/entrez/viewer.fcgi?db=protein&val=ANW96115.1)), AXE80_07425 ([ANW96116.1](http://www.ncbi.nlm.nih.gov/entrez/viewer.fcgi?db=protein&val=ANW96115.1)), AXE80_07310 ([ANW96098.1](http://www.ncbi.nlm.nih.gov/entrez/viewer.fcgi?db=protein&val=ANW96115.1)) and AXE80_07305 ([ANW96097.1](http://www.ncbi.nlm.nih.gov/entrez/viewer.fcgi?db=protein&val=ANW96115.1)); D1818_06650 (AXT50524.1) and D1818_06655 (AXT50525.1); Fp273 (AYC81238.1), Fp277 (AYC81239.1) and Fp279 (AYC81240.1); FNB79_00785 (QDO92576.1) from *Formosa sediminum* PS13; Fleli_2704 (AFM05060.1) from *Bernardetia litoralis* DSM6794; SVI_0379 ([BAJ00350.1](http://www.ncbi.nlm.nih.gov/entrez/viewer.fcgi?db=protein&val=BAJ00350.1)); Fda1 ([AAO00508.1](http://www.ncbi.nlm.nih.gov/entrez/viewer.fcgi?db=protein&val=AAO00508.1)), Fda2 ([AAO00509.1](http://www.ncbi.nlm.nih.gov/entrez/viewer.fcgi?db=protein&val=AAO00508.1)); Amino acids conserved for all known fucoidanases are indicated by the red background and the catalytic active site residues are indicated by a yellow triangle below the alignment. Amino acids of high conservation are indicated with red letters and encircled by borders in blue.


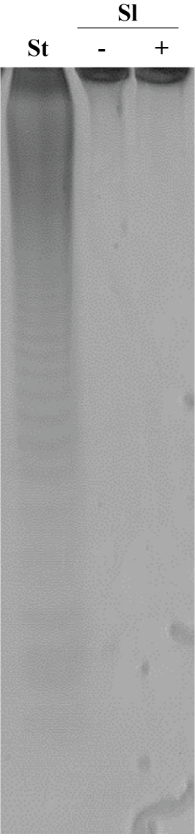


**Figure S2. Activity of Fhf2∆484 on fucoidans from *S. latissima*.** C-PAGE of -) the reaction of *S. latissima* fucoidan without Fhf2∆484 enzyme +) reaction with Fhf2∆484 enzyme and *S. latissima* fucoidan. (St) positive control, reaction of FFA2 on *F. evanescens* fucoidan.
